# Three new terpenoids from the fruit peels of *Citrus Hassaku* Yu.Tanaka

**DOI:** 10.1007/s11418-025-01983-7

**Published:** 2025-12-03

**Authors:** Daisuke Imahori, Takuya Muraoka, Tomoe Ohta, Tatsusada Yoshida, Hiroyuki Tanaka

**Affiliations:** 1 Department of Pharmacognosy and Kampo, Faculty of Pharmaceutical Sciences, Sanyo-Onoda City University, 1-1-1 Daigaku-dori, Sanyo-Onoda City, Yamaguchi 756-0884 Japan; 2 Kyoto Pharmaceutical University, Misasagi, Yamashina-ku, Kyoto, 607-8412 Japan; 3 Faculty of Pharmaceutical Sciences, Nagasaki International University, 2825-7 Huis Ten Bosch-Cho, Sasebo, Nagasaki, 859-3298 Japan

**Keywords:** Rutaceae, *Citrus hassaku*, Limonoids, Sesquiterpenoids, Anti-proliferative effects

## Abstract

*Citrus hassaku* Yu. Tanaka is a relatively less-known species within the genus. In this study, we aimed to isolate novel compounds from this species, elucidate their chemical structures via spectroscopic and physicochemical analyses, and evaluate their antiproliferative effects using the WST-8 assay. Two new limonoids, 1-acetyl-sphaerocarpainic acid I (**1**) and 1-acetyl-sphaerocarpain I (**2**), and a new eremophilane-type nor-sesquiterpenoid enantiomer, 12-nor-11*S*-hydroxy-11-hydronootkatone (**9**), were isolated from the peels of *C. hassaku*, together with eleven known compounds: deacetylnomilin (**3**), nomilin (**4**), methyl nomilinate (**5**), obacunone (**6**), limonin (**7**), ichangin (**8**), nootkatone (**10**), (+)-(4*R*,5*S*,7*R*)-13-hydroxynootkatone (**11**), umbelliferone (**12**), auraptene (**13**), and marmin (**14**). Among them, compound (**9**) exhibited weak antiproliferative effects against human glioblastoma U-251 MG cells.

**Graphical abstract:**

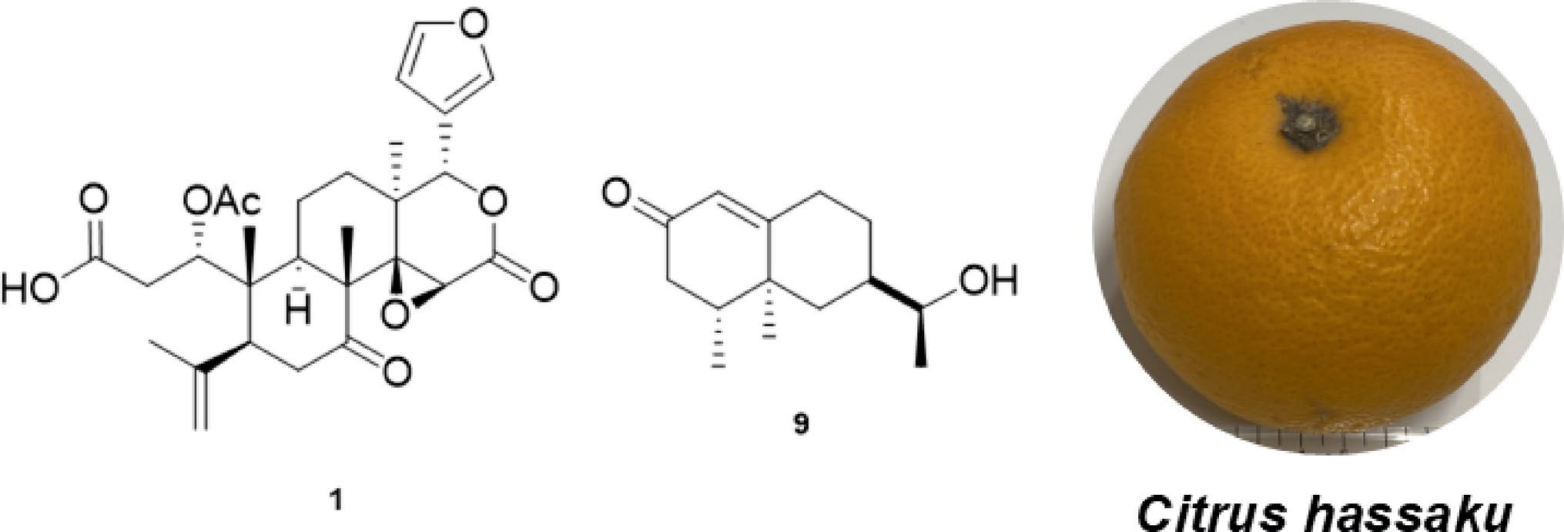

**Supplementary Information:**

The online version contains supplementary material available at 10.1007/s11418-025-01983-7.

## Introduction

The *Citrus* genus belonging to the *Rutaceae* family, comprises some of the most widely cultivated fruit crops worldwide [1], encompassing 40 different species [2]. *Citrus* fruits yield numerous value-added products, such as canned goods, juice, puree, essential oils, pickles, and vinegar [3]. They are rich sources of bioactive compounds such as flavonoids, carotenoids, limonoids, coumarins, and vitamin C [4,5]. Many of these compounds have demonstrated notable bioactivities, such as cytotoxic [6], antiviral [7], antimutagenic [8], and insect antifeedant [9] effects. *C. hassaku* Yu.Tanaka is one of the most popular *Citrus* species in Japan [10] and is assumed to be a hybrid of Kunenbo (*C. reticulata*) and other *Citrus* varieties [11]. Its fruits are consumed fresh, processed into juice, and used in natural medicine [10], reported to exhibit cancer chemopreventive activities [12], tyrosinase inhibitory activity [13], antiallergic activity [14], and cytotoxicity [15]. Phytochemical studies on *C. hassaku* have identified flavonoids [16], coumarins [17,18], sesquiterpenoids [15], and limonoids [19,20]. Sesquiterpenoids such as nootokatone and limonoids such as limonin have been reported to exert antitumor effects [21,22].

In this study, we isolated three new constituents from *C. hassaku* and revealed that one of them showed weak anti-proliferative effects against a human glioblastoma cell line.

## Results

### Isolation of 14 compounds from *C*. *hassaku*

**Fig. 1:**
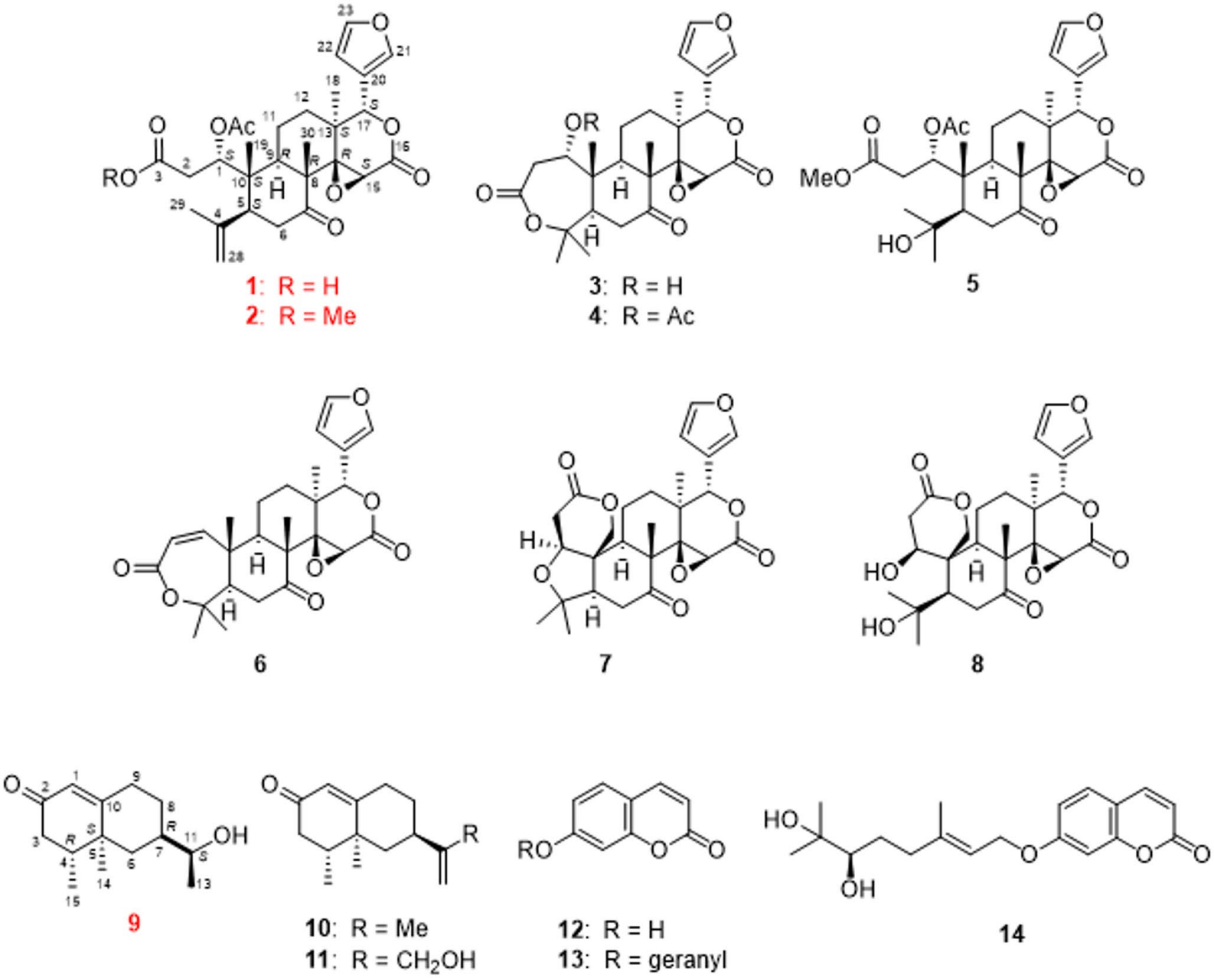
Skeletal (shorthand) structures of the compounds isolated from the peels of *C. hassaku* Yu.Tanaka

The methanol (MeOH) extract of the peels of *C. hassaku* was partitioned in ethyl acetate (EtOAc) and water (1:1), yielding an EtOAc (1.1%) soluble fraction and an aqueous layer. The aqueous layer was further extracted with *n*-butanol (BuOH) to obtain the *n*-BuOH-(1.5%) and H_2_O (10.0%) fractions. The EtOAc soluble fraction was subjected to normal- and reversed-phase silica gel column chromatography, followed by HPLC, affording two new limonoids—1-acetyl-sphaerocarpainic acid I (**1**, 0.00014%) and 1-acetyl-sphaerocarpain I (**2**, 0.00016%)—and one new eremophilane-type nor-sesquiterpenoid enantiomer, 12-nor-11*S*-hydroxy-11-hydronootkatone (**9**, 0.000046%). Furthermore, 11 known compounds were also isolated: deacetylnomilin (**3**, 0.00015%) [23], nomilin (**4**, 0.0032%) [23], methyl nomilinate (**5**, 0.00012%) [24], obacunone (**6**, 0.00008%) [25], limonin (**7**, 0.0049%) [26], ichangin (**8**, 0.00007%) [27], nootkatone (**10**, 0.0017%) [28], (+)-(4*R*,5*S*,7*R*)-13-hydroxynootkatone (**11**, 0.0001%) [29], umbelliferone (**12**, 0.00002%) [30], auraptene (**13**, 0.00016%) [31], and marmin (**14**, 0.008%) [30] (Fig. 1).

### Structures of 1-acetyl-sphaerocarpainic acid I, 1-acetyl-sphaerocarpain I, and 12-nor-11 S-hydroxy-11-hydronootkatone (**1**, **2**, and **9**)

**Fig. 2:**
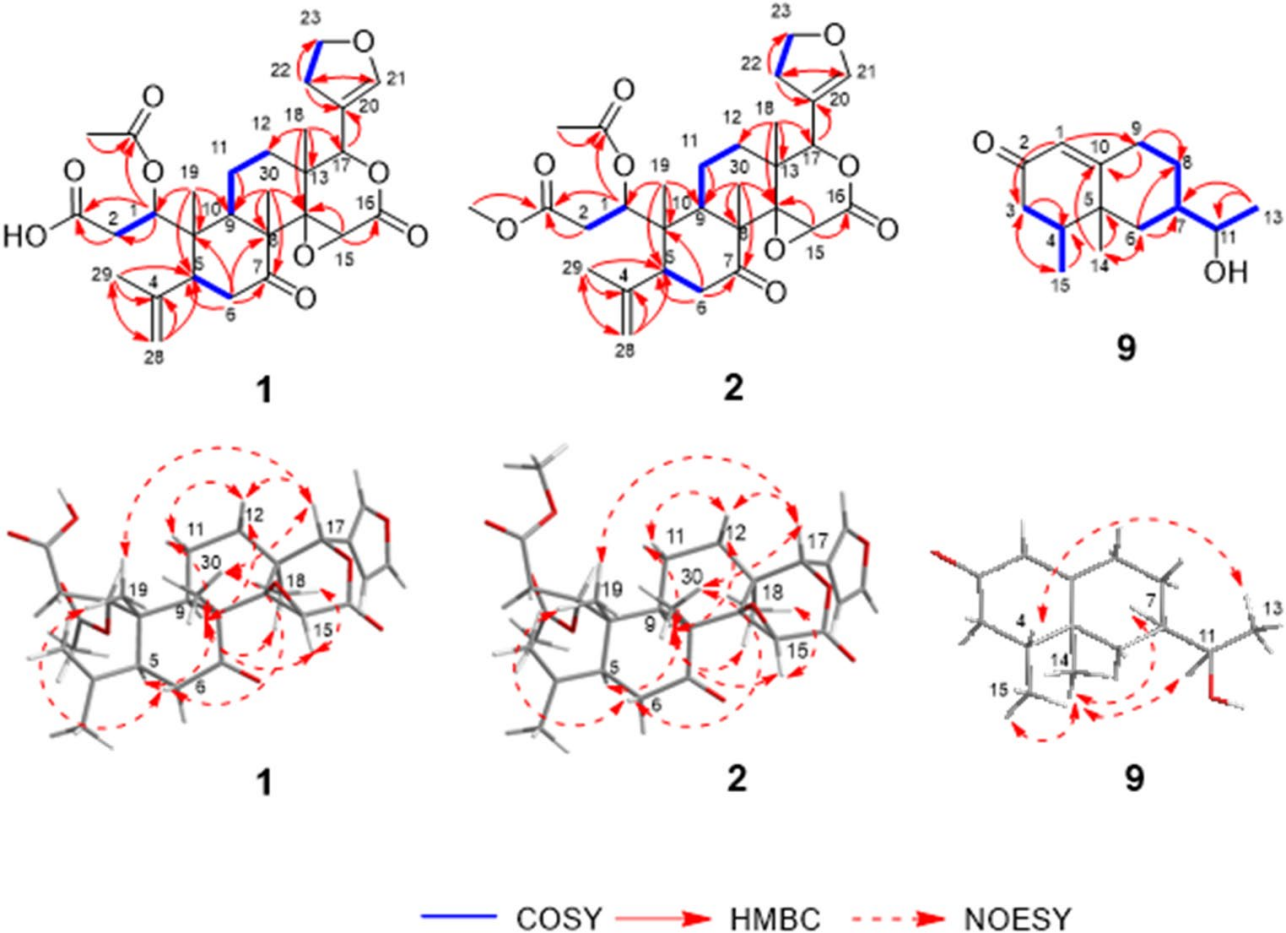
2D NMR and NOESY correlations for 1-acetyl-sphaerocarpainic acid I, 1-acetyl-sphaerocarpain I, and 12-nor-11*S*-hydroxy-11-hydronootkatone (**1**, **2**, and **9**)

**Fig. 3:**
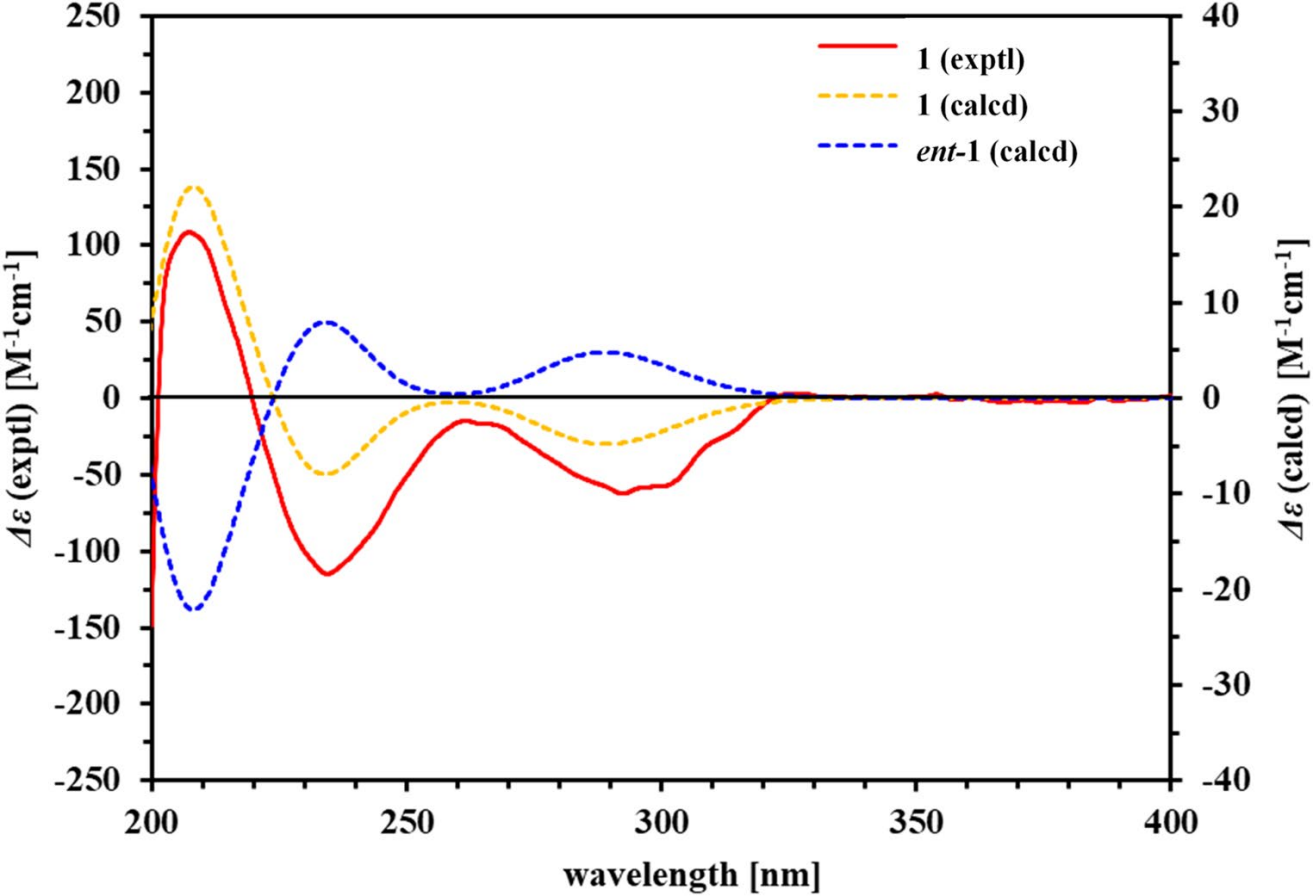
Comparison of experimental and calculated ECD spectra for the two possible enantiomers of **1** in MeOH (band width σ = 0.30 eV, shift = 12 nm)

1-Acetyl-sphaerocarpainic acid I (**1**) was isolated as a white amorphous powder with a negative optical rotation $\:{\left[a\right]}_{D}^{25}$–94.4 [MeOH]. Its molecular formula (C_28_H_34_O_9_) was determined using high-resolution (HR) ESI-MS and ^13^C NMR spectroscopy. The 1D NMR data (Table 1) of **1** indicated carbon skeletons similar to those of sphaerocarpain I [32], with the major difference being the presence of an acetoxy group (C-1). The ^1^H and ^13^C NMR spectra (CDCl_3_) exhibited characteristic signals of a limonoid moiety (Table 1), including four methyl groups [*δ*_H_ 1.09 (s, H-18), 1.15 (s, H-19), 1.82 (s, H-29), and 1.17 (s, H-30), each 3 H], an acetoxy group [*δ*_H_ 2.04 (s); *δ*_C_ 20.9 (OAc-1)], four methylene groups [*δ*_C_ 35.2 (C-2), 41.4 (C-6), 18.6 (C-11), 32.3 (C-12)], three methine groups bearing an oxygen function [*δ*_C_ 75.2 (C-1), 53.1 (C-15), 78.1 (C-17), a furan ring [*δ*_H_ 7.41 (br s, H-21), 6.36 (s, H-22), and 7.40 (br s, H-23), each 1 H], two ester carbonyl groups [*δ*_C_ 174.6 (C-3), 166.9 (C-16), and a ketone group [*δ*_C_ 209.0 (C-7)]. In **1**, the C-16 and C-17 carbon signals appear at *δ*_C_ 166.9 and 78.1, respectively (Table 1, in chloroform-*d*). In contrast, open-ring limonoids such as limonoate A-ring lactone [33] and nomilinoate A-ring lactone [33] exhibit downfield-shifted C-16 signals (*δ*_C_ 173.7 and 171.5) and upfield-shifted C-17 signals (*δ*_C_ 72.5 for both) (Table S1, in methanol-*d*_3_), consistent with ring cleavage and altered electronic environments. This trend is consistent with previous reports on ring-seco limonoids, where ring opening leads to deshielding at C-16 due to increased electron-withdrawing effects and shielding at C-17 due to loss of ring strain. This comparative data (Table 1, Fig. S2.11, and Table S1) provides additional evidence for the closed-ring structure of compound **1**, independent of HMBC correlations. The positions of each functional group were determined via COSY and HMBC spectroscopy (Fig. 2). Namely, long-range correlations were observed between the following proton and carbon pairs: H-1 with C-3, C-9, and C-OAc-1 (*δ*_C_ 170.2); H-2 with C-3; H-6 with C-5, C-7, C-8, and C-10; H-11 with C-9; H-15 with C-14 and C-16; H-17 with C-112, C-13, C-14, C-18, C-20, C-21, and C-22; H-18 with C-12, C-13, C-14, and C-17; H-19 with C-1, C-5, C-9, and C-10; H-21 with C-20, C-22, and C-23; H-22 with C-20, C-21, and C-23; H-23 with C-20, C-21, and C-22; H-28 with C-4, C-5, and C-29; H-29 with C-4, C-5, and C-28; H-30 with C-7, C-8, C-9, and C-14; and OAc-1 with C-OAc-1 (*δ*_C_ 170.2). 1-Acetyl-sphaerocarpainic acid I (**1**) is a limonoid in which the A ring is cleaved and has an isopropenyl group attached to the C-5 side chain. The methyl protons H-29 (*δ*_H_ 1.82) and H-28 (*δ*_H_ 4.89 and 5.08) also showed cross-peaks corresponding to C-5 in the HMBC spectra, indicating the position of the isopropenyl group (C-4, C-28, and C-29) at C-5. Furthermore, an HMBC correlation between H-1 and the carbon resonance at *δ*_C_ 170.2 assigned the acetoxy to C-1. Several limonoids with the same functional group, such as agleduline J [34], dysomollide E [35], and polystanin A [36] have been reported. The relative configuration of the ring system was determined from the NOESY spectrum (Fig. 2), revealing cross peaks of H-5 with H-9; H-6*b* with H-19 and H-30; H-9 with H-11*α*, H-12*α*, and H-18; H-11*b* with H-12*b*; H-15 with H-9 and H-18; and H-17 with H-12*b*, H-19, and H-30. Such results indicated that H-2*α*, 5, 6*α*, 9, 11*α*, 12*α*, 15, 18, and 28*α* are on one side of each molecule and that 2*b*, 6*b*, 11*b*, 12*b*, 17, 19, 28*b* and 30 are on the opposite side. The absolute configuration of the ring system was determined using the calculated ECD curve (Fig. 3A). The calculated ECD spectra of **1** were identical to those of the experimental data, whereas the calculated ECD spectra of *ent*-**1** displayed the opposite Cotton effects. These results suggested that the absolute configurations were 5*S*, 8*R*, 9*R*, 10*S*, 13*S*, 14*R*, 15*S*, and 17*S*. However, due to the conformational flexibility of the side chain, NOESY correlations were insufficient to unambiguously assign the stereochemistry at C-1. To resolve this, **2** (C-3 methoxy analogue of **1**) was converted to methyl nomilinate (**5**) [24] via Mukaiyama hydration [37,38] (Figure S2.12). Based on biosynthetic studies in *Citrus* [6,22], and the stereochemical assignment of methyl nomilinate (**5**) [24], the absolute configuration at C-1 in **2** was determined to be 1 S. Given the identical stereochemical environment around C-1 in **1**, the same configuration was reasonably assigned. Thus, the absolute configuration of **1** was established as 1* S*, 5*S*, 8*R*, 9*R*, 10*S*, 13*S*, 14*R*, 15*S*, and 17*S*. Based on these findings, the chemical structure of **1** is presented in Fig. 1.

**Table 1:** ^13^C (150 MHz) and ^1^H (600 MHz) NMR data for compounds **1**, **2**, and **9** in chloroform-*d*

Position	1		2		9	
	*δ*_C_	*δ*_H_ (*J* in Hz)	*δ*_C_	*δ*_H_ (*J* in Hz)	*δ*_C_	*δ*_H_ (*J* in Hz)
1	75.2	5.57 (br d, 9.6)	75.4	5.53 (dd, 1.8, 10.8)	124.6	5.76 (s)
2	35.2	*a* 2.39 (m) *b* 2.74 (d-like, 14.4)	35.4	*a* 2.37 (m) *b* 2.70 (dd, 1.8, 14.5)	199.6	
3	174.6		171.0		42.1	2.26 (2 H, m)
4	143.6		143.5		40.5	1.99 (m)
5	50.9	2.67 (br s)	50.8	2.67 (m)	39.0	
6	41.4	*a* 2.33 (m) *b* 3.03 (t-like, 13.2)	41.4	*a* 2.33 (d-like, 5.2, 14.8) *b* 3.03 (t-like, 12.0)	40.7	*a* 2.15 (d, 13.1) *b* 0.94 (d, 13.1)
7	209.0		209.1		40.3	1.73 (m)
8	52.9		52.9		28.7	*a*1.84–1.88 (m) *b* 1.25–1.26 (m)
9	43.8	2.18 (br s)	43.7	2.19 (d, 11.7)	32.7	*a* 2.47 (m) *b* 2.37 (m)
10	44.5		44.4		170.6	
11	18.6	*a* 1.67 (m) *b* 2.20 (m)	18.6	*a* 1.67 (m) *b* 2.22 (m)	71.7	3.59 (t-like, 6.0)
12	32.3	*a* 1.41 (m) *b* 1.79 (m)	32.3	*a* 1.42 (m) *b* 1.79 (m)		
13	37.2		37.2		20.8	1.22 (d, 7.2)
14	65.3		65.3		16.9	1.09 (s)
15	53.1	3.73 (s)	53.1	3.74 (s)	15.0	0.99 (d, 6.6)
16	166.9		166.9			
17	78.1	5.44 (s)	78.1	5.44 (s)		
18	20.9	1.09 (s)	20.9	1.09 (s)		
19	15.9	1.15 (s)	16.0	1.14 (s)		
20	120.2		120.3			
21	140.9	7.41 (br s)	140.9	7.41 (br s)		
22	109.8	6.36 (s)	109.8	6.36 (s)		
23	143.1	7.40 (br s)	143.1	7.40 (br s)		
28	117.5	*a* 4.89 (s) *b* 5.08 (s)	117.4	*a* 4.90 (s) *b* 5.08 (s)		
29	22.5	1.82 (s)	22.5	1.82 (s)		
30	16.9	1.17 (s)	16.9	1.17 (s)		
1-OAc	170.2		169.9			
	20.9	2.04 (s)	20.9	2.04 (s)		
3-OMe			52.2	3.64 (s)		

1-Acetyl-sphaerocarpain I (**2**) was isolated as a white amorphous powder with a negative optical rotation $\:{\left[a\right]}_{D}^{25}$–130.8 [MeOH]. Its molecular formula (C_29_H_36_O_9_) was determined via HR-ESI-MS and ^13^C NMR spectroscopy. The 1D and 2D NMR spectra of **2** were generally similar to those of **1**, suggesting that **2** is also a nomilin-type limonoid, with the major difference being the presence of a methoxy group (C-3). The ^1^H and ^13^C NMR spectra (CDCl_3_) exhibited characteristic signals of a methoxy group [*δ*_H_ 3.64 (s); *δ*_C_ 52.2] (Table 1). The HMBC correlation between H-3-OMe and *δ*_C_ 171.0 assigned the methoxy group to C-3 (Fig. 2). The relative configuration of the ring system in **2** was determined by NOESY analysis, showing the same spatial arrangement as **1** (Fig. 2). The absolute configuration of the ring system was established by comparison of the experimental CD spectrum of **2** with that of **1** (Fig. S2.10). For the flexible side chain, the same approach as used for **1**, conversion to methyl nomilinate (**5**) [24] followed by stereochemical inference based on biosynthetic precedent [6,22], was applied. These results confirmed that **2** shares the same absolute configuration as **1**, namely 1*S*, 5*S*, 8*R*, 9*R*, 10*S*, 13*S*, 14*R*, 15*S*, and 17*S*. Based on this evidence, the chemical structure of **2** is presented in Fig. 1.

**Fig. 4:**
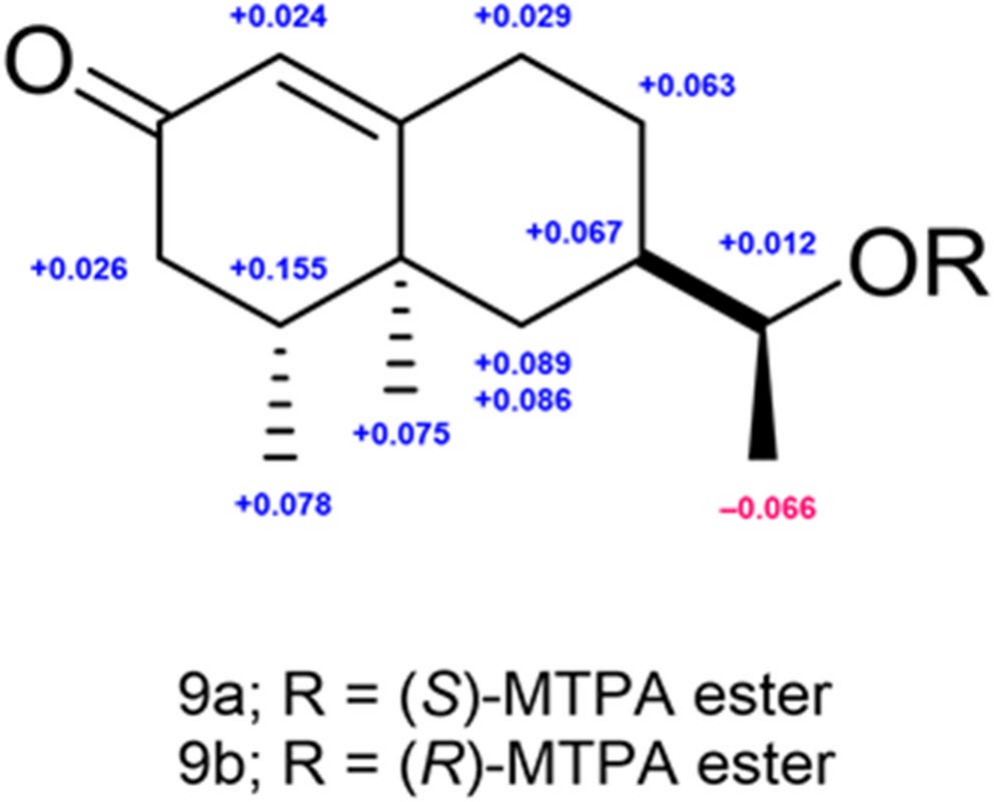
*Δd*_H_ values (*d*_*S*−ester_–*d*_*R*−ester_) for the diastereomeric MTPA esters of compound **9** (600 MHz, pyridine-*d*₅, ppm)

12-Nor-11*S*-hydroxy-11-hydronootkatone (**9**) was isolated as a white amorphous powder with a positive optical rotation $\:{\left[a\right]}_{D}^{25}$ +9.57 [MeOH]. Its molecular formula (C_14_H_22_O_2_) was determined via HR-ESI-MS and ^13^C NMR spectroscopy. The 1D NMR data (Table 1) of **9** were characteristic of eremophilane-type sesquiterpenoids and closely resembled those of nootkatone (**10**). Compound **9** has a tertiary methyl group [*δ*_H_ 1.09 (3 H, s)], two secondary methyl groups [*δ*_H_ 0.99 (3 H, d, *J* = 6.6 Hz)], a 1-hydroxyethyl group [*δ*_H_ 3.59 (1 H, t-like, *J* = 6.0 Hz); *δ*_C_ 71.7 and 1.22 (3 H, d, *J* = 6.0 Hz); *δ*_C_ 20.8], and a ketone [*δ*_C_ 199.6]. The positions of each functional group were determined form the COSY and HMBC spectra (Fig. 2). Long-range correlations (**9**) were observed between the following proton and carbon pairs: H-1 with C-3 and C-9; H-3 with C-4; H-6 with C-7, C-8, C-10, C-11, and C-14; H-9 with C-8 and C-10; H-13 with C-7 and C-11; and H-15 with C-3. The relative configuration of the ring system was determined *via* analysis of the NOESY spectrum (Fig. 2), revealing cross peaks of H-7 with H-14; H-14 with H-15. The absolute configuration of the ring system was further confirmed by comparison of the experimental CD spectrum of **9** with that of nootkatone (**10**), which shares a similar chromophore and known stereochemistry (Fig. S5.1). In contrast, the stereochemistry of the side chain was independently determined using modified Mosher’s method [39]: **9** was derivatized with (*R*)- and (*S*)-*a*-methoxy-*a*-(trifluoromethyl)phenylacetyl chlorides [(*R*)- and (*S*)-MTPA] to yield the diastereomeric esters **9a** and **9b**, respectively. Comparison of the ¹H NMR chemical shifts of **9a** and **9b** in pyridine-*d*₅ revealed positive *Δd* values for H-11, H-13, H-14, and H-15 (Figs. 4, S5.2, and S5.3), consistent with an 11*S* configuration. Based on comprehensive spectroscopic data and stereochemical analyses, including 2D NMR, CD, and modified Mosher’s method [39], the absolute configuration of **9** was established as 4*R*, 5*S*, 7*R*, and 11*S* (Fig. 1).

### Evaluation of anti-proliferative effects

The antiproliferative effects of the fruit extracts and the isolated compounds (**1**–**14**) were evaluated in human glioma U-251 MG cells using the WST-8 assay, where WST-8 is reduced by an NADH-dependent cellular oxidoreductase to form formazan (maximum absorbance 450 nm), which is directly proportional to the number of viable cells [21]. The fruit extracts exhibited anti-proliferative effects against U-251 MG cells (Fig. S6). Among all compounds (30 µM) tested, only compound **9** showed a measurable reduction in U-251 MG cell proliferation when compared with control cells (Fig. S7), decreasing cell proliferation to 75.3 ± 6.1% (Fig. S7). For comparison, adriamycin (1.7 µM) reduced cell proliferation to 55.0 ± 1.88% (Fig. S6).

## Discussion

*Citrus* fruits are rich sources of bioactive compounds, including flavonoids, carotenoids, limonoids, sesquiterpenoids, and coumarins [4,21]. Sesquiterpenoids, including nootokatone and limonoids, including limonin, have been reported to exert antitumor effects [21,22]. Although some studies have examined the constituents of *C. hassaku* fruits, few have investigated their antiproliferative effects. The primary objective of this study was to isolate these compounds and evaluate their anti-proliferative activity. Our results revealed that *C. hassaku* contains terpenoids, such as limonin and nootkatone, consistent with previous reports [15,19]. Limonoids are unusual triterpenes owing to their extensively rearranged biosynthetic scaffolds [6]. At least two main scaffold modifications are proposed to be conserved in Meliaceae and Rutaceae plant families: a C-30 methyl shift of the protolimonoid scaffold (apo-rearrangement), and the conversion of the hemiacetal ring of melianol into a mature furan ring with the concomitant loss of a C4 carbon side-chain [40]. Additional modifications that are specific to Rutaceae then yield nomilin-type intermediates [6,22,40]. In this study, the carbon numbering of compounds **1** and **2** was assigned based on biosynthetic logic and structural homology to nomilin-type limonoids, rather than following the numbering used in previously reported sphaerocarpain I [32]. This approach reflects the conserved scaffold rearrangements and facilitates comparison with other limonoids derived from *Citrus* species. The numbering scheme is consistent with recent biosynthetic studies that trace limonoid formation from melianol-type precursors (Fig. 1). Furthermore, the fruit extracts exhibited antiproliferative effects against human glioma U-251 MG cells (Fig. S6), in agreement with previous findings on human glioma U-373 MG cells [15]. The content of bioactive compounds typically depends on various factors such as genomic differences, climatic conditions, cultural practices, harvest time, as well as industrial and extraction systems [4]. Among the isolated compounds, compound **9** showed weak antiproliferative effects against U-251 MG cells. However, given the limitations of the included studies, further investigations regarding the biological activities of these components are required, particularly in vivo toxicity assessments and clinical trials, to determine effective doses and validate their therapeutic potential.

## Conclusion

In summary, two new limonoids, 1-acetyl-sphaerocarpainic acid I and 1-acetyl-sphaerocarpain I (**1** and **2**), and one new eremophilane-type norsesquiterpenoid enantiomer, 12-nor-11*S*-hydroxy-11-hydronootkatone (**9**), were isolated from the peels of *C. hassaku*. The chemical structures of the new compounds were elucidated based on chemical and physicochemical analyses. The absolute configurations of **1** and **2** were established by comparing experimental and predicted ECD data. Compound **9** exhibited weak inhibitory activity against the proliferation of U-251 MG cells.

## Methods

### General experimental procedures

Specific rotations were measured using a P-2200 digital polarimeter (*l* = 5 cm; JASCO, Tokyo, Japan). FTIR spectra were recorded on a JASCO FT/IR-4600 Fourier transform infrared spectrometer. UV spectra were obtained using a Shimadzu UV-1850 UV/vis spectrophotometer (Shimadzu, Kyoto, Japan). ECD spectra were recorded using a JASCO J-500CH spectrometer. ESI-MS was performed using a 6470 Triple Quad LC/MS (Agilent Technologies, CA, USA), while HR-ESI-MS was performed using a JMS-T100LP AccuTOF LC-plus 4G atmospheric pressure ionization high-resolution time-of-flight mass spectrometer (JEOL, Tokyo, Japan). ^1^H, ^13^C, and 2D NMR spectra were recorded using a JEOL JNM-ECZ 600R spectrometer. Normal-phase silica gel column chromatography was performed on a Wakogel^®^ 60 N (FUJIFILM Wako Pure Chemical, Osaka, 63–212 μm). Reversed-phase silica gel column chromatography was performed on a C_18_-OPN (Nacalai Tesque, Kyoto, Japan, 75 μm). TLC was performed using TLC plates pre-coated with 60F_254_ silica gel (Merck, Darmstadt, Germany; 0.25 mm, ordinary phase) and Merck RP-18 F_254_S silica gel (0.25 mm, reversed phase). HPLC was performed using an SPD-10Avp UV-vis detector (Shimadzu, Kyoto, Japan). The COSMOSIL 5C_18_-AR-II (Nacalai Tesque, 250 × 4.6 mm i.d., 250 × 10 mm i.d.), COSMOSIL Cholester (Nacalai Tesque, 250 × 4.6 mm i.d. and 250 × 10 mm i.d.), and YMC-Triart PFP (YMC, Kyoto, Japan, 250 × 4.6 mm i.d. and 250 × 10 mm i.d.) columns were used for analytical and preparative purposes. The solvent ratios were based on the volume. 3D models of the isolated compounds were obtained using ChemDraw 20.1.1 software.

### Plant material

*C. hassaku* fruits were purchased from the Japan Agricultural Cooperative (JA) farmers’ market in Yamaguchi Prefecture (Japan), supplied by a local farmer, in May 2022 (voucher specimen ID SOCU-2022-19). Total DNA was extracted from plant samples, and PCR amplification and sequencing were performed by Rizo Inc. (Sample number; PD0151, Tsukuba, Japan)15. The obtained nucleotide sequences were analyzed using the BLAST tool available at the National Center for Biotechnology Information (NCBI) website (https://blast.ncbi.nlm.nih.gov/Blast.cgi). BLAST analysis was also conducted by Rizo Inc. Sequences were queried against the NCBI nucleotide collection (nr/nt) database using default parameters. The top hits with the highest sequence identities and lowest E values were used for taxonomic identification. Plant species were identified based on sequence similarity. The voucher specimens were deposited in the herbarium of the Department of Pharmacognosy, Faculty of Pharmaceutical Sciences, Sanyo-Onoda City University, Yamaguchi, Japan. The plant material was identified by Prof. Dr. Hiroyuki Tanaka (PhD) (Department of Pharmacognosy and Kampo, Faculty of Pharmaceutical Sciences, Sanyo-Onoda City University). Fruits, peels, and leaves were dried in the shade and stored.

### Extraction and isolation

Dried peels of *C. hassaku* (5.0 kg) were extracted three times with MeOH under reflux for 3 h. Evaporation of the solvent provided the MeOH extract (629.9 g), which was partitioned into ethyl acetate/water (1:1) to obtain the EtOAc-soluble fraction (55.8 g) and the aqueous layer. The aqueous layer was further extracted with *n*-BuOH to afford the *n*-BuOH- (74.6 g) and H_2_O- (499.5 g, 9.99%) fractions. The EtOAc soluble fraction was subjected to normal phase silica gel column chromatography [2.0 kg, hexane/CHCl_3_ (20:1 → 5:1 → 1:1 → 1:5) → CHCl_3_ → CHCl_3_/MeOH (1:0 → 200:1 → 100:1 → 50:1 → 10:1 → 7:1 → 5:1 → 1:1)] to give eight fractions (Fr. CHEA1–8). Fr. CHEA 5 and 6 (9.1 g) were further separated via reversed phase silica gel column chromatography [450 g, MeOH/H_2_O (4:6 → 5:5 → 6:4 → 7:3 → 8:2 → 9:1)] to afford ten subfractions (Fr. CHEA5, 6 − 1–5, 6–10). Compound **13** (400 mg) was precipitated as a yellow amorphous powder from subfraction CHEA5,6–7. Subfraction CHEA5,6 − 2 (400 mg) was purified *via* HPLC [H_2_O/MeCN/AcOH (65:35:0.3)] to obtain 14 subfractions (Fr. CHEA5,6-2-1–5,6-2-14). Subfraction CHEA5,6-2-1 (12 mg) was purified *via* HPLC [H_2_O/MeCN/AcOH (65:35:0.3)] to afford compound **9** (2.3 mg) as a white amorphous powder. Subfraction CHEA5,6 − 3 (1 g) was purified *via* HPLC [H_2_O/MeCN/AcOH (65:35:0.3)] to yield compounds **4** (7.5 mg), **7** (245 mg), and **11** (46.3 mg) as white amorphous powders and 16 subfractions (Fr. CHEA5,6-3-1–5,6-3-16). Subfraction CHEA5,6-3-7 (101 mg) was purified *via* HPLC [H_2_O/MeCN/AcOH (75:25:0.3)] to yield compound **12** (13.8 mg) as a white amorphous powder. Subfraction CHEA5,6-3-16 (162 mg) was purified *via* HPLC [H_2_O/MeCN/AcOH (45:55:0.3)] to yield compounds **1** (6.2 mg), **2** (5.7 mg), and **5** (8.3 mg) as white amorphous powders. Subfraction CHEA5,6 − 4 (400 mg) was purified *via* HPLC [H_2_O/MeCN/AcOH (65:35:0.3)] to afford compound **6** (4.0 mg) as a white amorphous powder. Subfraction CHEA5,6–6 (600 mg) was purified *via* HPLC [H_2_O/MeCN/AcOH (50:50:0.3)] to yield compound **10** (82.5 mg) as a white amorphous powder. Fr. CHEA7 (7.3 g) was further separated via reversed phase silica gel column chromatography [370 g, MeOH/H_2_O (2:8 → 3:7 → 4:6 → 5:5 → 6:4 → 7:3 → 8:2 → 9:1)] to yield 11 subfractions (Fr. CHEA7-1–7–11). Compound **14** (242 mg) was precipitated as a yellow liquid from subfraction CHEA7-8. Subfraction CHEA7-7 (509 mg) was purified *via* HPLC [H_2_O/MeCN/AcOH (70:30:0.3)] to afford compounds **3** (7.5 mg) and **8** (3.5 mg) as white amorphous powders.

### 1-Acetyl-sphaerocarpainic acid I (**1**)

White amorphous powder; $\:{\left[a\right]}_{D}^{25}$–94.4 (*c* 0.1, MeOH); FT-IR (ATR) *v*_max_ 1032, 1346, 1718, 2359, 2866, 2972, 3680 cm^– 1^; UV (MeCN) *λ*_max_ (log*ε*) 201.5 nm (3.93); CD (MeOH) *λ*_max_ nm (*Δε*): 207 nm (108.4), 235 nm (–114.9), 292 nm (–62.4); ^1^H NMR (CDCl_3_, 600 MHz) and ^13^C NMR (150 MHz) spectroscopic data see Table 1; HR-ESI-MS *m*/*z*: 537.20951 (Calcd for C_28_H_34_O_9_N_a_ [M + Na]^+^ *m*/*z*: 537.20950).

### 1-Acetyl-sphaerocarpain I (**2**)

White amorphous powder; $\:{\left[a\right]}_{D}^{25}$–130.8 (*c* 0.1, MeOH); FT-IR (ATR) *v*_max_ 1033, 1346, 1729, 2358, 2866, 2973, 3681 cm^– 1^; UV (MeCN) *λ*_max_ (log*ε*) 202.5 nm (4.12) ; CD (MeOH) *λ*_max_ nm (*Δε*): 202.9 nm (53.2), 232 nm (–50.5), 292 nm (–28.3); ^1^H NMR (CDCl_3_, 600 MHz) and ^13^C NMR (150 MHz) spectroscopic data see Table 1; HR-ESI-MS *m*/*z*: 551.22769 (Calcd for C_29_H_36_O_9_N_a_ [M + Na]^+^*m*/*z*: 551.22515).

### 12-Nor-11 S-hydroxy-11-hydronootkatone (**9**)

Colorless oil; $\:{\left[a\right]}_{D}^{25}$+9.57 (*c* 0.1, MeOH); FT-IR (ATR) *v*_max_ 1022, 1056, 1346, 1586, 2361, 2971, 3696 cm^−1^; UV (MeCN) *λ*_max_ (log*ε*) 199.5 nm (3.27), 234.0 nm (3.12), 291.0 nm (2.57); CD (MeOH) *λ*_max_ nm (*Δε*): 237 nm (65.3); ^1^H NMR (CDCl_3_, 600 MHz) and ^13^C NMR (150 MHz) spectroscopic data see Table 1; HR-ESI-MS *m*/*z*: 245.15212 (Calcd for C_28_H_34_O_9_N_a_ [M + Na]^+^ *m*/*z*: 245.15120).

### Mosher ester analysis of 12-Nor-11 S-hydroxy-11-hydronootkatone (**9**)

In an NMR sample micro-bottom tube (5 mm diameter), 120 µl of pyridine-*d*_5_ was mixed with 1.0 mg (40 mM) of compound **9**, followed by the addition of 5.0 µl of (*R*)-MTPA chloride (TCI, Tokyo, Japan, 160 mM). The resulting reaction mixture was stirred at RT for 30 min to produce the (*S*)-MTPA ester **9a**. An identical procedure was followed to obtain the (*R*)-MTPA ester **9b** from (*S*)-MTPA chloride (TCI, 160 mM). **9a**: ^1^H NMR (600 MHz, pyridine-*d*₅, ppm) *δ*_H_ 5.793 (1H, s, H-1), 2.191 (2 H, m, H-3), 1.941 (1H, m, H-4), 1.643 (1H, m, H-6), 0.991 (1H, m, H-6), 1.748 (1H, m, H-7), 1.850 (1H, m, H-8), 0.793–0.835 (1H, m, H-8), 2.111 (1H, m, H-9), 2.2615 (1H, m, H-9), 4.989 (1H, m, H-11), 1.171 (1H, d, *J* = 6.0, H-13), 0.816 (1H, s, H-14), 0.705 (1H, d, *J* = 6.6, H-15). **9b**: ^1^H NMR (600 MHz, pyridine-*d*₅, ppm) *δ*_H_ 5.769 (1H, d, *J* = 1.8, H-1), 2.165 (2 H, m, H-3), 1.786 (1H, m, H-4), 1.554 (1H, m, H-6), 0.905 (1H, m, H-6), 1.681 (1H, m, H-7), 1.787 (1H, m, H-8), 0.701 (1H, m, H-8), 2.082 (1H, m, H-9), 2.219–2.291 (1H, m, H-9), 4.977 (1H, m, H-11), 1.237 (1H, d, *J* = 6.0, H-13), 0.741 (1H, s, H-14), 0.627 (1H, d, *J* = 6.0, H-15).

### Calculation of theoretical ECD spectra

The initial geometries of the conformers of 1*S*,5*S*,8*R*,9*R*,10*S*,13*S*,14*R*,15*S*,17*S*-(**1**) were generated and then geometrically optimized *in vacuum* using the Merck molecular force field (MMFF) as implemented in Spartan ’18 [41]. Low-energy conformers with Boltzmann distributions >1% were further optimized at the *w*B97X-D/def2-TZVP level using density functional theory (DFT). To ensure that none of the conformers exhibited imaginary frequencies and obtain enthalpies (*H*), including zero-point energy (ZPE) corrections, normal mode analyses were performed at the same level [42,43]. Distinctive low-energy conformers (Fig. S4) with Boltzmann distributions >1% were subjected to ECD calculations using time-dependent density functional theory (TD-DFT) at the *w*B97X-D/ma-TZVPP level (the def2-TZVPP basis set with *s* and *p* diffuse basis functions on non-hydrogen atoms [44,45]). All DFT and TD-DFT calculations were performed using an integral equation formalism polarizable continuum model (IEFPCM) in MeOH with Gaussian 16 [46]. The resulting rotatory strengths of the lowest 30 excited states for each conformer were converted into Gaussian-type curves with half bands using SpecDis v1.71 [47]. The final calculated ECD spectra were obtained via Boltzmann-distribution correction of the conformers, based on their relative enthalpies, including the ZPE correction (*ΔH*).

### Cell culture

U-251 MG cells (IFO50288, Japanese Collection of Research Bioresources Cell Bank, Osaka, Japan) were cultured in Dulbecco’s modified Eagle’s medium (DMEM) with low glucose (FUJIFILM Wako Pure Chemical Industries, Osaka, Japan) supplemented with 10% fetal bovine serum (FBS: FUJIFILM Wako Pure Chemical) and 5% penicillin-streptomycin solution (FUJIFILM Wako Pure Chemical) under a 5% CO_2_ atmosphere at 37 ℃.

### WST-8 assay

Cell proliferation was assessed using a cell counting kit 8 (CCK-8; FUJIFILM Wako Pure Chemical) according to the manufacturer’s instructions. Cells were seeded at a density of 2.5 × 10^3^ cells/100 µl per well in 96-well cell culture plates (Coster 3596; Corning, NY, USA). After approximately 24 h, the cells were treated with adriamycin (FUJIFILM Wako Pure Chemical) or the isolated compounds (30 µM) for 24 h. A CCK-8 solution containing WST-8 [2-(2-methoxy-4-nitrophenyl)-3-(4-nitrophenyl)-5-(2,4disulfophenyl)-2*H*-tetrazolium, monosodium salt] (10 µl) was added to the plates and incubated in a CO_2_ incubator for 3 h. The absorbance was measured at 450 and 620 nm using a microplate reader (Multiskan FC; Thermo Fisher Scientific, Waltham, MA, USA).

### Statistical analysis

Statistical analyses were performed using GraphPad Prism 8.43 software. Differences between treatment groups were evaluated using Dunnett’s test, with **P < 0.01 considered statistically significant compared with DMSO-treated cells.

## Supplementary Information

Below is the link to the electronic supplementary material.

Supplementary Material 1. Supplementary InformationExperimental details: 1H, 13C, 2D NMR, CD spectra and optimized geometries, minimum value of frequency, relative enthalpies including ZPE correction, and Boltzmann distributions of conformers of the new compounds.
