# Supplementary material for: Three new terpenoids from the fruit peels of Citrus Hassaku Yu.Tanaka
Source: J Nat Med. 2025 Dec 3;80(1):230–9. doi: 10.1007/s11418-025-01983-7 (PMC12847175; doi:10.1007/s11418-025-01983-7)
Supplement: Supplementary file 1 — Supplementary Material 1. Supplementary InformationExperimental details: 1H, 13C, 2D NMR, CD spectra and optimized geometries, minimum value of frequency, relative enthalpies including ZPE correction, and Boltzmann distributions of conformers of the new compounds. [file 11418_2025_1983_MOESM1_ESM.docx]

**Three new terpenoids from the fruit peels *Citrus hassaku* Yu.Tanaka**

**Authors**

Daisuke Imahori^a^, Takuya Muraoka^a^, Tomoe Ohta^b^, Tatsusada Yoshida^c^, and Hiroyuki Tanaka^a,^*

**Affiliations**

^a^Faculty of Pharmaceutical Sciences, Sanyo-Onoda City University, 1-1-1 Daigaku-dori, Yamaguchi 756-0884, Japan.

^b^Kyoto Pharmaceutical University, Misasagi, Yamashina-ku, Kyoto 607-8412, Japan

^c^Faculty of Pharmaceutical Sciences, Nagasaki International University, 2825-7 Huis Ten Bosch-Cho, Sasebo, Nagasaki 859-3298, Japan.

**Correspondence**

*Hiroyuki Tanaka, Ph. D. Professor

Department of Pharmacognosy and Kampo, Faculty of Pharmaceutical Sciences, Sanyo-Onoda City University, 1-1-1 Daigaku-dori, Sanyo-Onoda City, 756-0884, Yamaguchi, Japan

Phone: +810836398574, Fax: +810836398574, E-mail: htanaka@rs.socu.ac.jp

**List of Supplementary material**

**Figure S1.1.1. ^1^H NMR spectrum of** **1-acetyl-sphaerocarpainic acid I (1)** **in CDCl_3_.**

**Figure S1.1.2. ^1^H NMR** **spectrum of 1-acetyl-sphaerocarpainic acid I (1)** **in CDCl_3_.**

**Figure S1.1.3. ^1^H NMR spectrum of 1-acetyl-sphaerocarpainic acid I (1)** **in CDCl_3_.**

**Figure S1.2. ^13^C NMR spectrum of 1-acetyl-sphaerocarpainic acid I (1)** **in CDCl_3_.**

**Figure S1.3.1. HMQC spectrum of 1-acetyl-sphaerocarpainic acid I (1)** **in CDCl_3_.**

**Figure S1.3.2. HMQC spectrum of 1-acetyl-sphaerocarpainic acid I (1)** **in CDCl_3_.**

**Figure S1.4.1. HMBC spectrum of 1-acetyl-sphaerocarpainic acid I (1)** **in CDCl_3_.**

**Figure S1.4.2. HMBC spectrum of 1-acetyl-sphaerocarpainic acid I (1)** **in CDCl_3_.**

**Figure S1.5. COSY spectrum of 1-acetyl-sphaerocarpainic acid I (1)** **in CDCl_3_.**

**Figure S1.6. NOESY spectrum of 1-acetyl-sphaerocarpainic acid I (1)** **in CDCl_3_.**

**Figure S1.7. IR spectrum of 1-acetyl-sphaerocarpainic acid I (1).**

**Figure S1.8. UV spectrum of 1-acetyl-sphaerocarpainic acid I (1).**

**Figure S1.9. MS spectrum of 1-acetyl-sphaerocarpainic acid I (1).**

**Figure S2.1.1. ^1^H NMR spectrum of 1-acetyl-sphaerocarpain I (2)** **in CDCl_3_.**

**Figure S2.1.2. ^1^H NMR spectrum of 1-acetyl-sphaerocarpain I (2)** **in CDCl_3_.**

**Figure S2.1.3. ^1^H NMR spectrum of 1-acetyl-sphaerocarpain I (2)** **in CDCl_3_.**

**Figure S2.2. ^13^C NMR spectrum of 1-acetyl-sphaerocarpain I (2)** **in CDCl_3_.**

**Figure S2.3.1. HMQC spectrum of 1-acetyl-sphaerocarpain I (2)** **in CDCl_3_.**

**Figure S2.3.2. HMQC spectrum of 1-acetyl-sphaerocarpain I (2)** **in CDCl_3_.**

**Figure S2.4.1. HMBC spectrum of 1-acetyl-sphaerocarpain I (2)** **in CDCl_3_.**

**Figure S2.4.2. HMBC spectrum of 1-acetyl-sphaerocarpain I (2)** **in CDCl_3_.**

**Figure S2.5. COSY spectrum of 1-acetyl-sphaerocarpain I (2)** **in CDCl_3_.**

**Figure S2.6. NOESY spectrum of 1-acetyl-sphaerocarpain I (2)** **in CDCl_3_.**

**Figure S2.7. IR spectrum of 1-acetyl-sphaerocarpain I (2).**

**Figure S2.8. UV spectrum of 1-acetyl-sphaerocarpain I (2).**

**Figure S2.9. MS spectrum of 1-acetyl-sphaerocarpain I (2).**

**Figure S2.10. CD spectrum of 1-acetyl-sphaerocarpain I (2).**

**Figure S2.11. Skeletal (shorthand) structures of 1-acetyl-sphaerocarpainic acid I (1) and 1-acetyl-sphaerocarpain I (2), limonoate A-ring lactone^27^, and nomilinoate A-ring lactone^27^.**

**Figure S2.12. Synthetic Procedure for the Conversion of Compound 2 to Methyl Nomilinate (5) via Mukaiyama Hydration.**

**Figure S3.1.1. ^1^H NMR spectrum of 12-nor-11S-hydroxy-11-hydronootkatone (9)** **in CDCl_3_.**

**Figure S3.1.2. ^1^H NMR spectrum of 12-nor-11S-hydroxy-11-hydronootkatone (9)** **in CDCl_3_.**

**Figure S3.2. ^13^C NMR spectrum of 12-nor-11S-hydroxy-11-hydronootkatone (9)** **in CDCl_3_.**

**Figure S3.3. HMQC spectrum of 12-nor-11S-hydroxy-11-hydronootkatone (9)** **in CDCl_3_.**

**Figure S3.4.1. HMBC spectrum of 12-nor-11S-hydroxy-11-hydronootkatone (9)** **in CDCl_3_.**

**Figure S3.4.2. HMBC spectrum of 12-nor-11S-hydroxy-11-hydronootkatone (9)** **in CDCl_3_.**

**Figure S3.5. COSY spectrum of 12-nor-11S-hydroxy-11-hydronootkatone (9)** **in CDCl_3_.**

**Figure S3.6. NOESY spectrum of 12-nor-11S-hydroxy-11-hydronootkatone (9)** **in CDCl_3_.**

**Figure S3.7. IR spectrum of 12-nor-11S-hydroxy-11-hydronootkatone (9).**

**Figure S3.8. UV spectrum of 12-nor-11S-hydroxy-11-hydronootkatone (9).**

**Figure S3.9. MS spectrum of 12-nor-11S-hydroxy-11-hydronootkatone (9).**

Figure S4. Optimized geometries, the minimum value of frequency, relative enthalpies including the ZPE correction, and Boltzmann distributions of conformers of 1-acetyl-sphaerocarpainic acid I (1).

Figure S5.1. CD spectra of 12-nor-11*S*-hydroxy-11-hydronootkatone (9) and nootkatone (10).

Figure S5.2. ^1^H NMR spectrum of 9a in pyridine-*d*_5._

Figure S5.3. ^1^H NMR spectrum of 9b in pyridine-*d*_5_.

Figure S6. Anti-proliferative effects evaluation of adriamycin by WST-8 assay.

Figure S7. Anti-proliferative effects evaluation of the isolated compounds (1–14) by WST-8 assay.

**Table S1. ^13^C (100 MHz) and ^1^H (400 MHz) NMR (in methanol-*d*_3_) data for limonoate A-ring lactone and nomilinoate A-ring lactone^27^.**


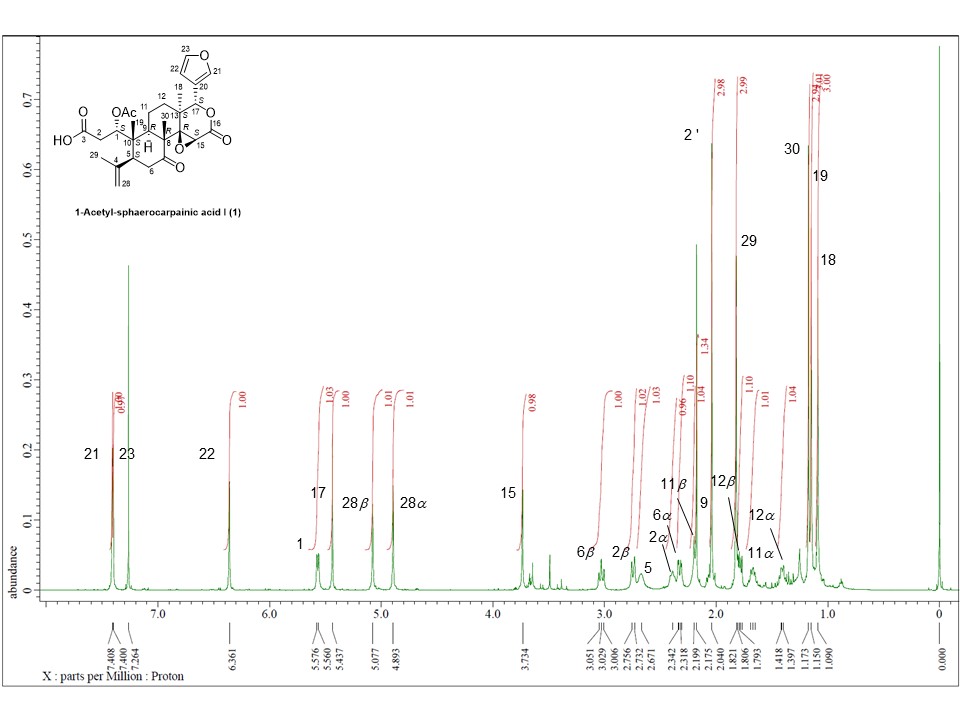


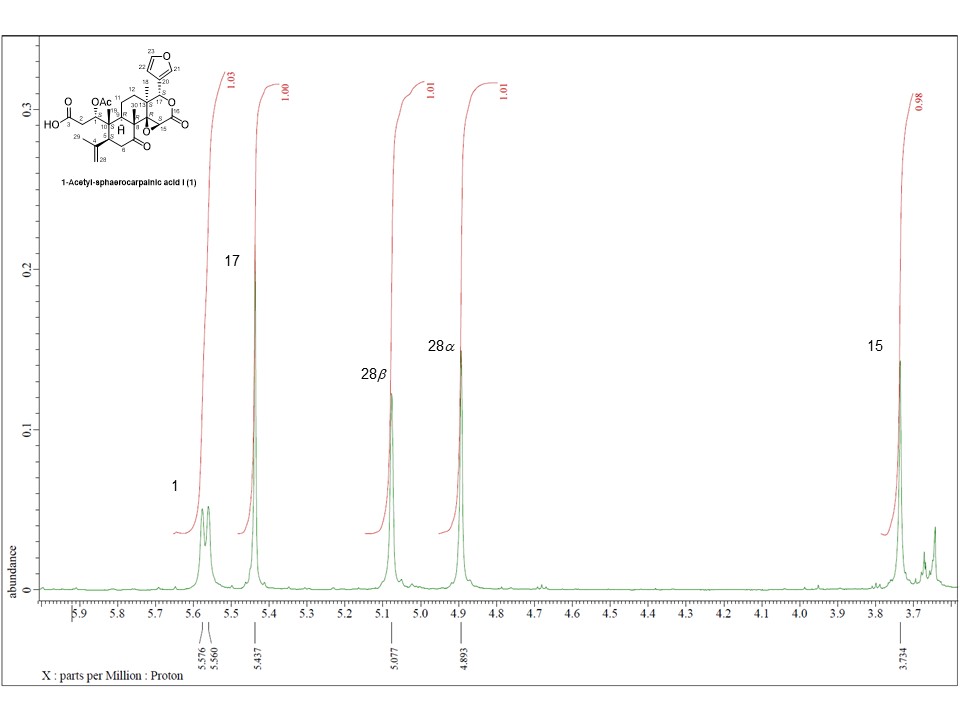
**Figure S1.1.1. ^1^H NMR spectrum of 1-acetyl-sphaerocarpainic acid I (1)** **in CDCl_3_.**

**Figure S1.1.2. ^1^H NMR spectrum of 1-acetyl-sphaerocarpainic acid I (1)** **in CDCl_3_.**


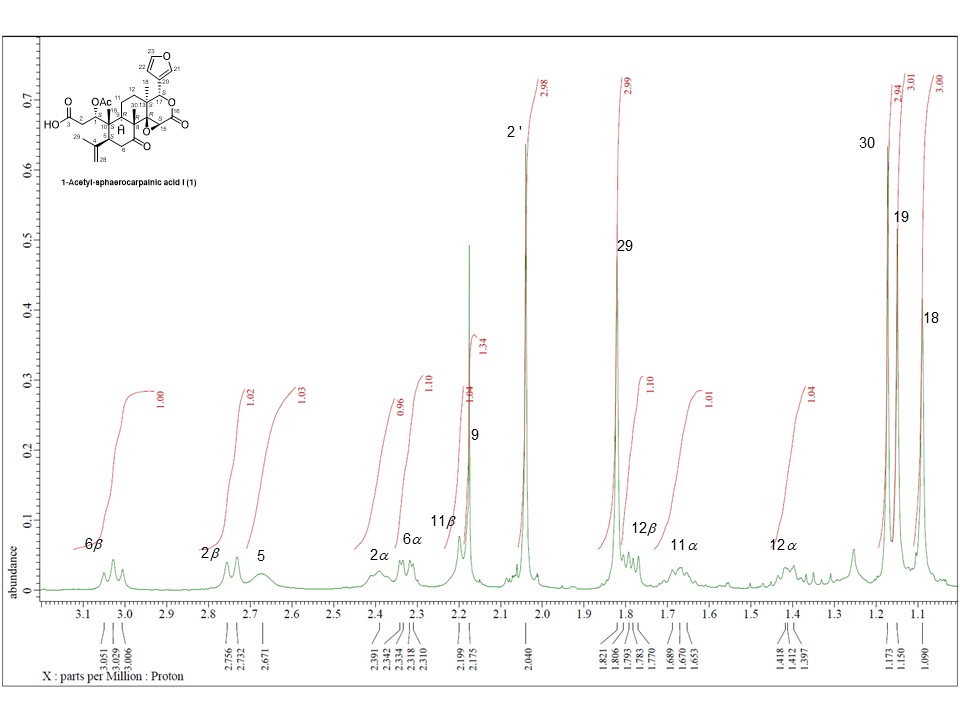


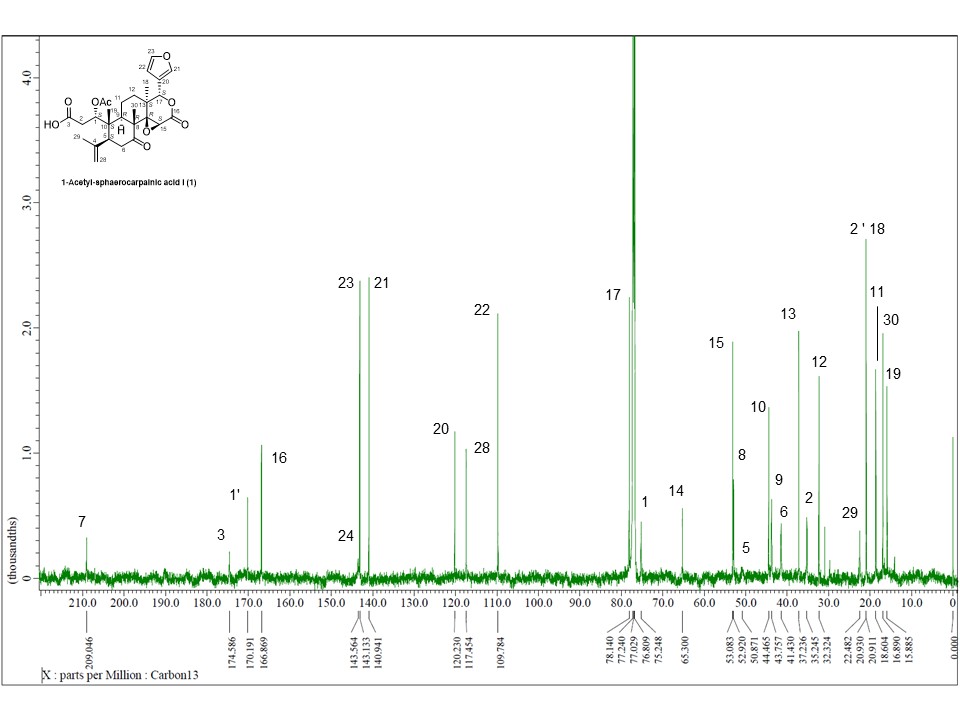
**Figure S1.1.3. ^1^H NMR spectrum of 1-acetyl-sphaerocarpainic acid I (1)** **in CDCl_3_.**

**Figure S1.2. ^13^C NMR spectrum of 1-acetyl-sphaerocarpainic acid I (1)** **in CDCl_3_.**


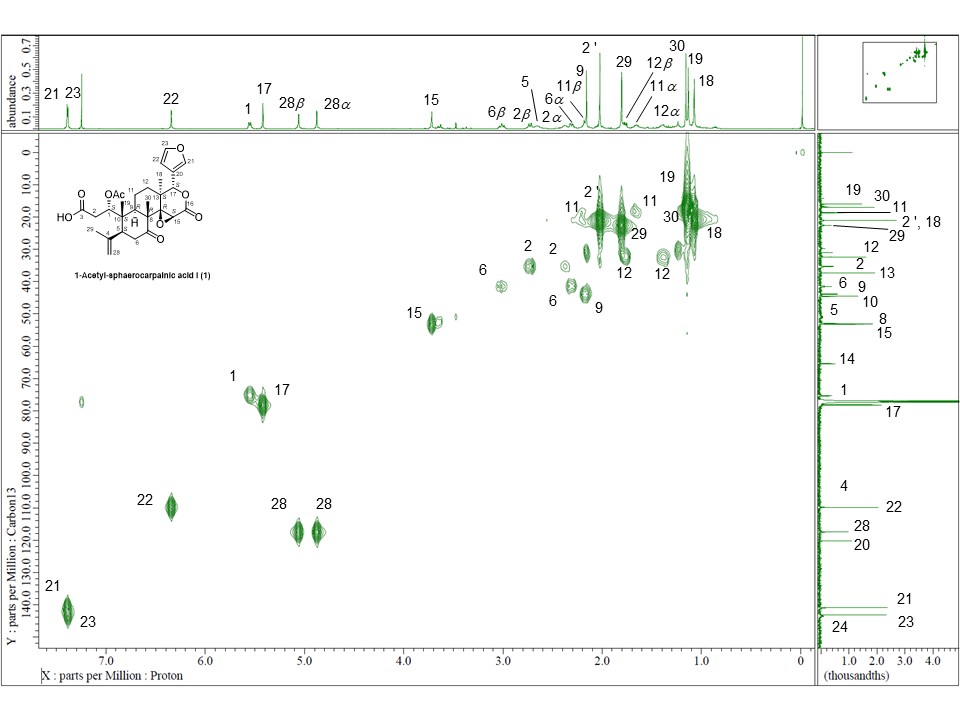


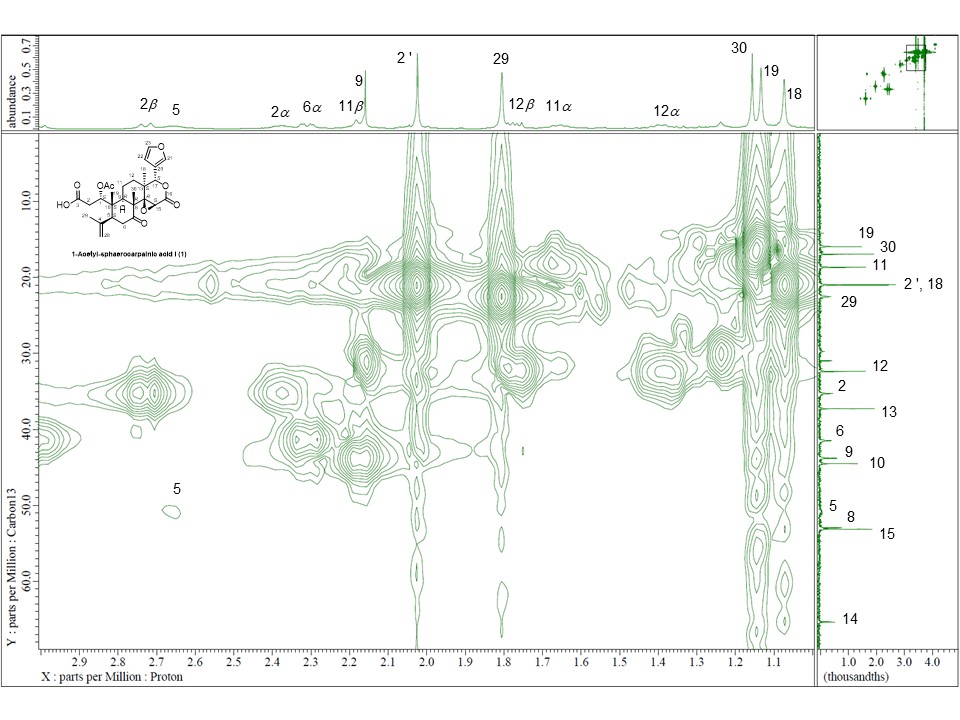
**Figure S1.3.1. HMQC spectrum of 1-acetyl-sphaerocarpainic acid I (1)** **in CDCl_3_.**

**Figure S1.3.2. HMQC spectrum of 1-acetyl-sphaerocarpainic acid I (1)** **in CDCl_3_.**


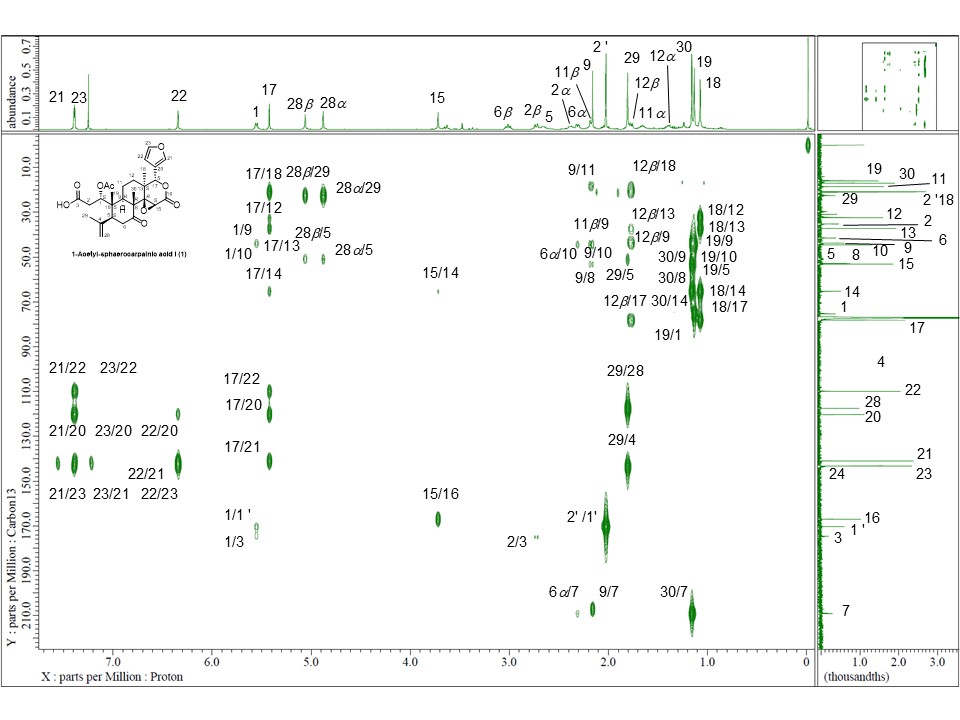


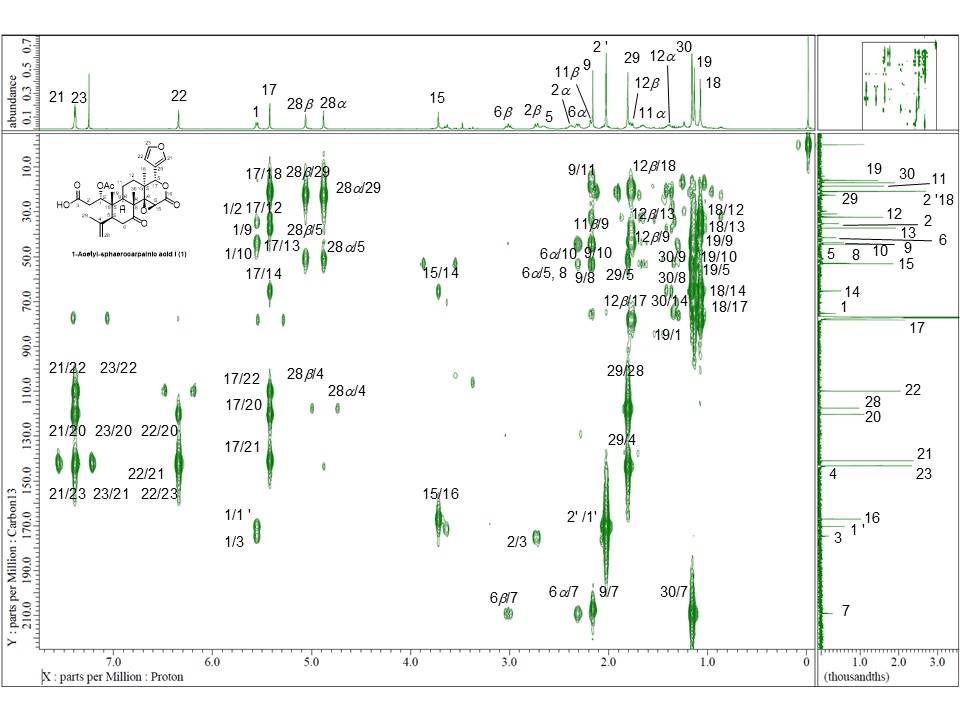
**Figure S1.4.1. HMBC spectrum of 1-acetyl-sphaerocarpainic acid I (1)** **in CDCl_3_.**

**Figure S1.4.2. HMBC spectrum of 1-acetyl-sphaerocarpainic acid I (1)** **in CDCl_3_.**


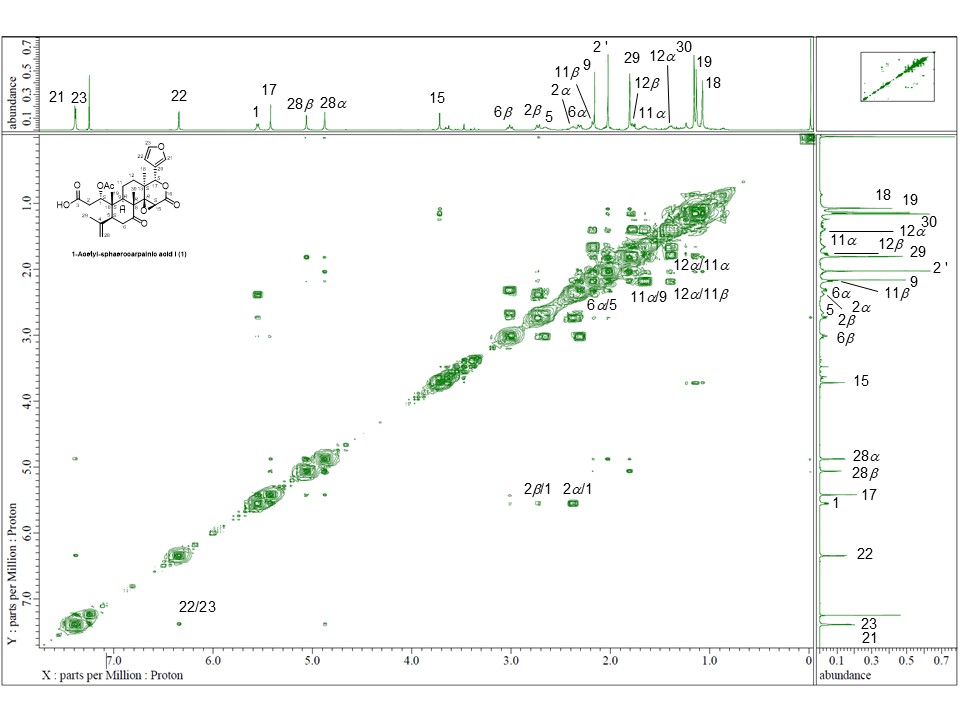


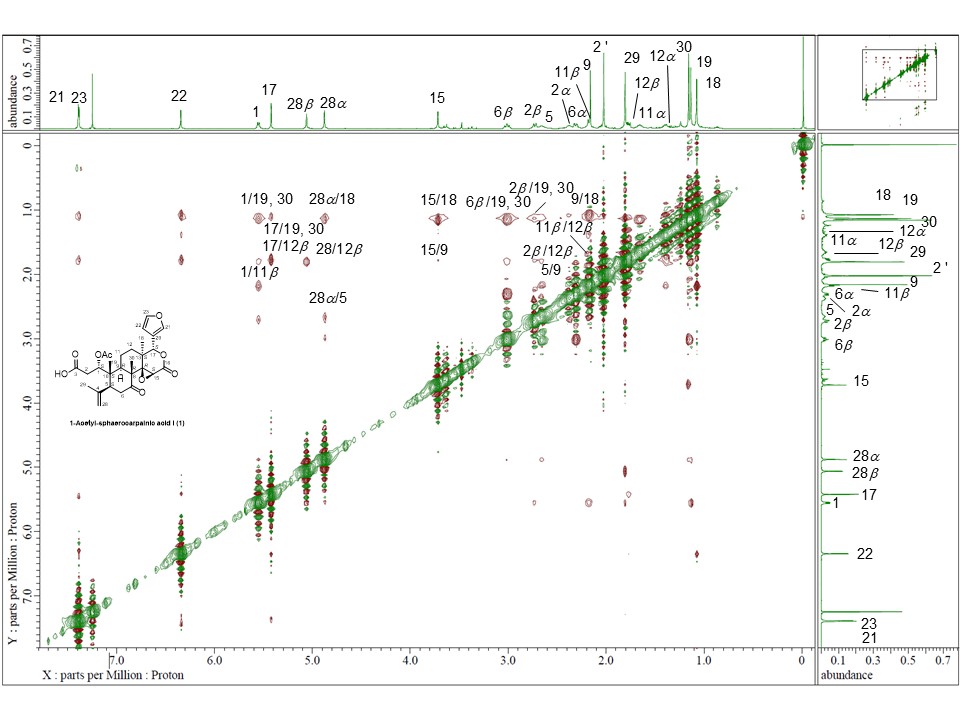
**Figure S1.5. COSY spectrum of 1-acetyl-sphaerocarpainic acid I (1)** **in CDCl_3_.**

**Figure S1.6. NOESY spectrum of 1-acetyl-sphaerocarpainic acid I (1)** **in CDCl_3_.**


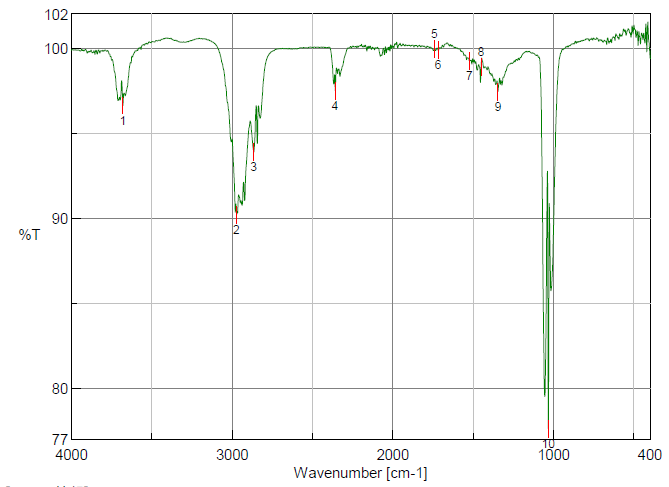


**Figure S1.7. IR spectra of 1-acetyl-sphaerocarpainic acid I (1).**


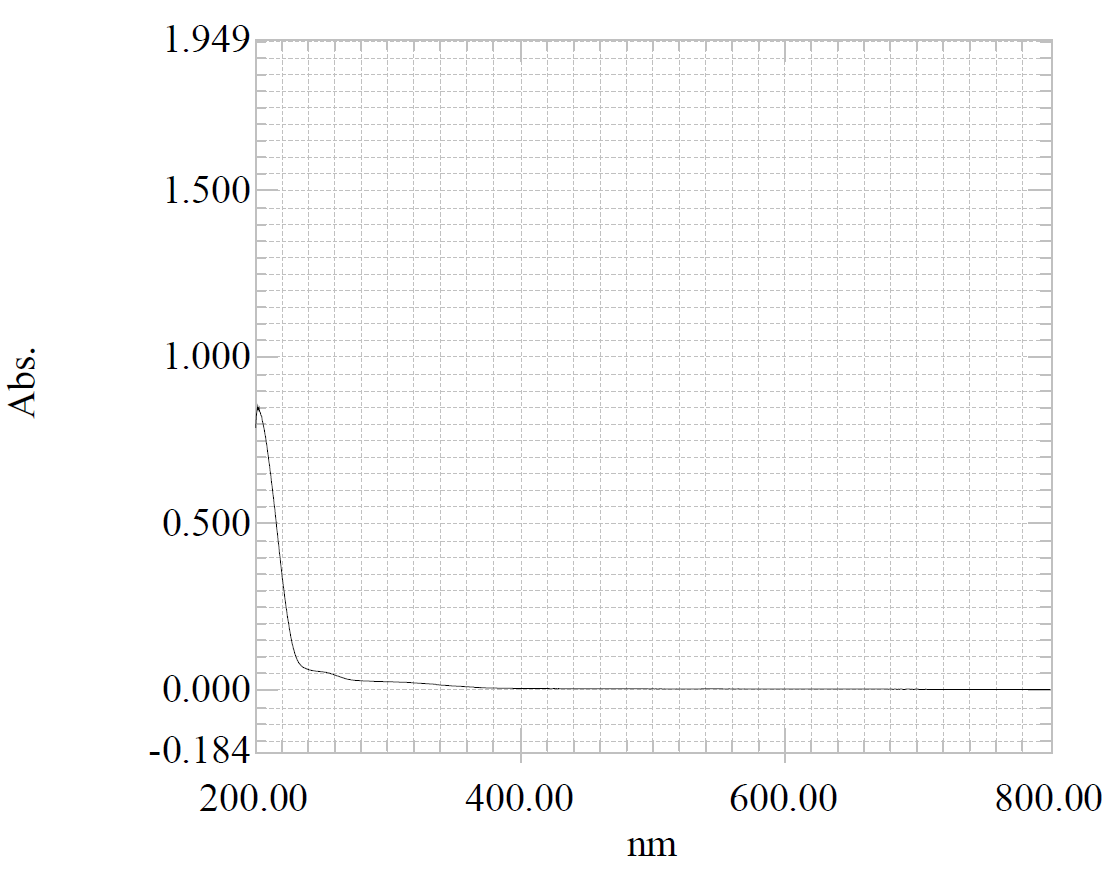


**Figure S1.8. UV spectra of 1-acetyl-sphaerocarpainic acid I (1).**


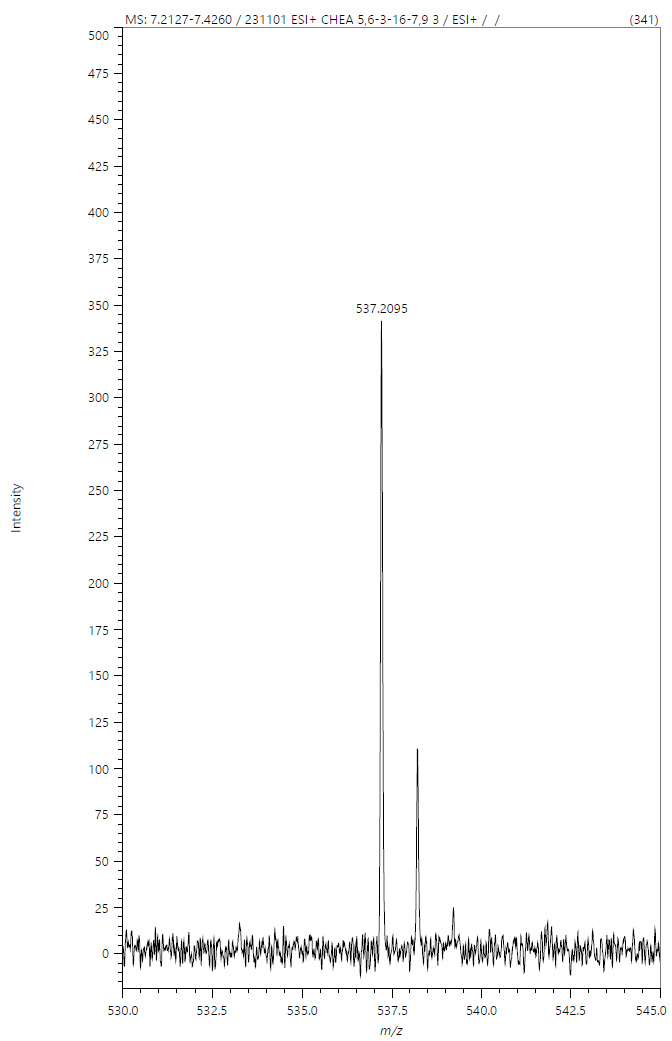


**Figure S1.9. MS spectra of 1-acetyl-sphaerocarpainic acid I (1).**


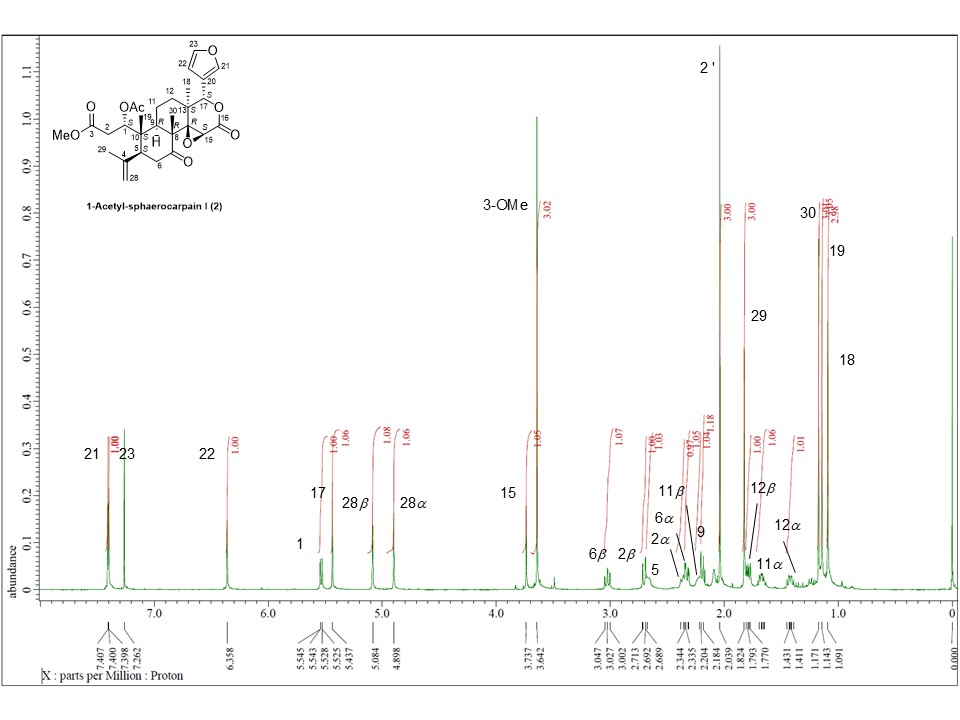


**Figure S2.1.1. ^1^H NMR spectrum of 1-acetyl-sphaerocarpain I (2)** **in CDCl_3_.**


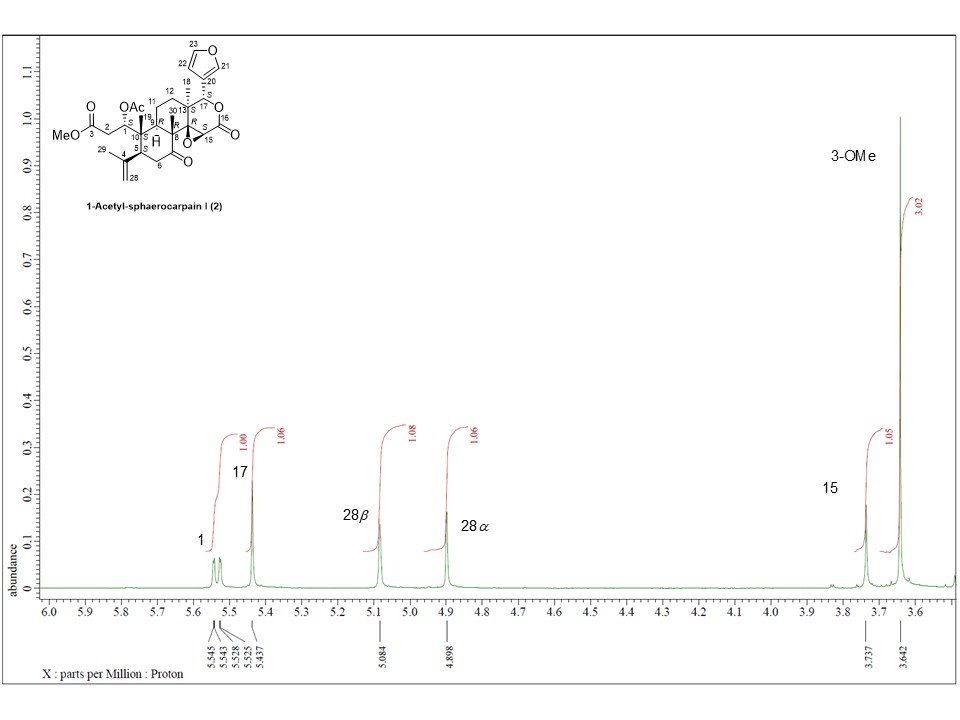


**Figure S2.1.2. ^1^H NMR spectrum of 1-acetyl-sphaerocarpain I (2)** **in CDCl_3_.**


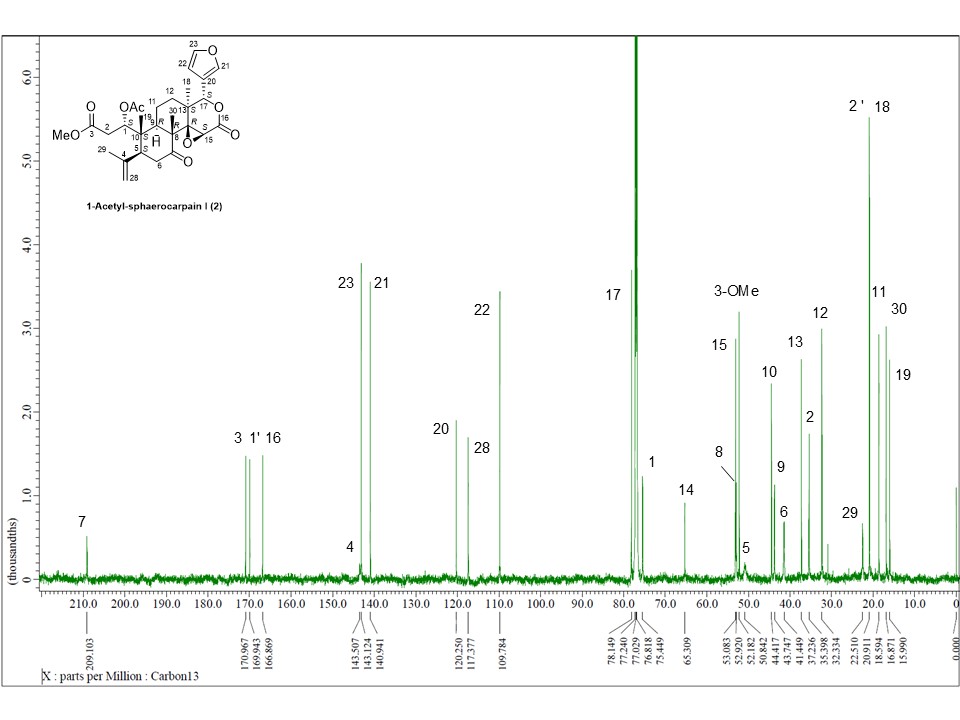

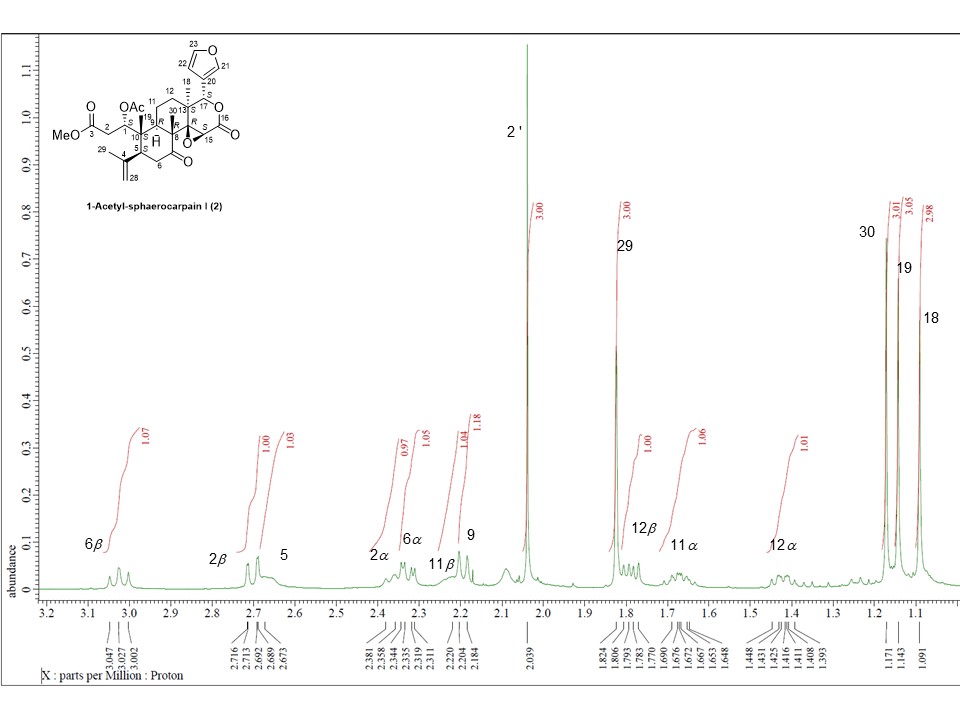


**Figure S2.1.3. ^1^H NMR spectrum of 1-acetyl-sphaerocarpain I (2)** **in CDCl_3_.**

**Figure S2.2. ^13^C NMR spectrum of 1-acetyl-sphaerocarpain I (2)** **in CDCl_3_.**


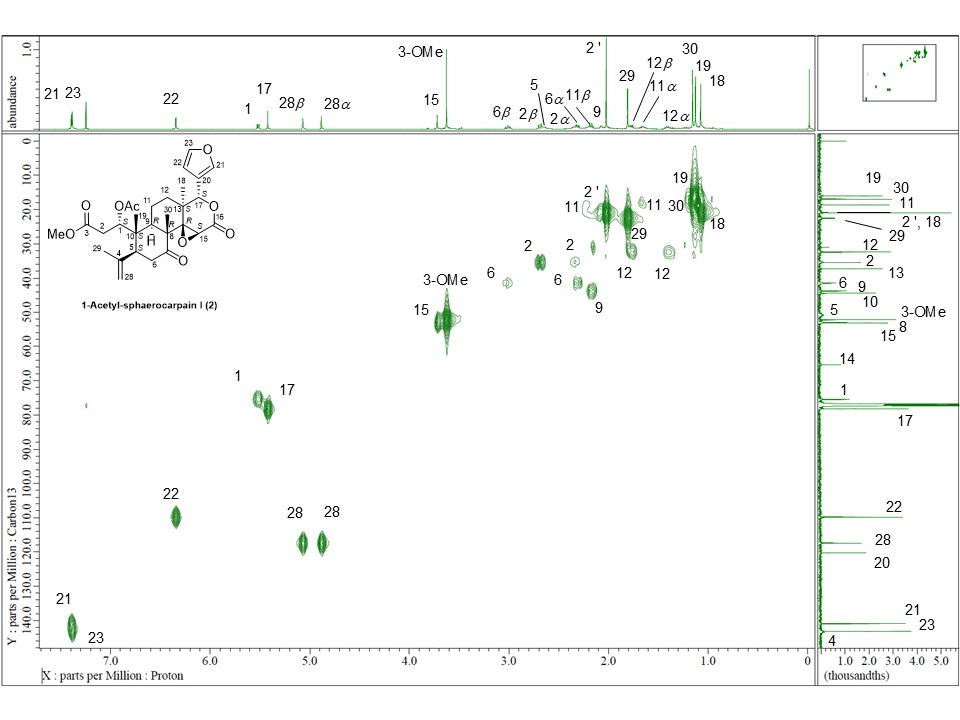


**Figure S2.3.1. HMQC spectrum of 1-acetyl-sphaerocarpain I (2)** **in CDCl_3_.**


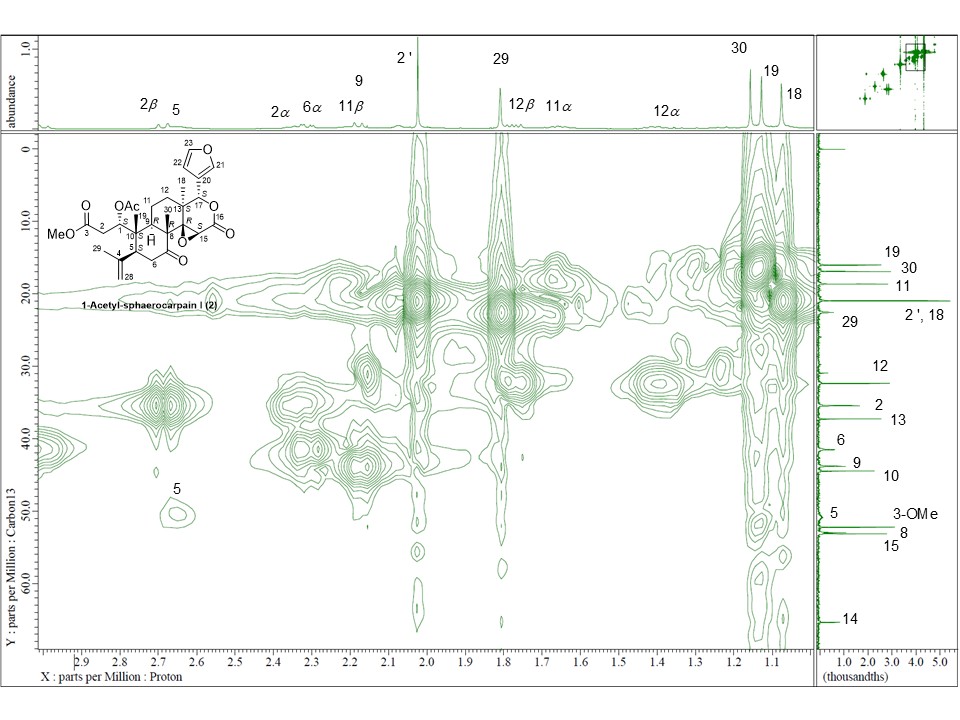


**Figure S2.3.2. HMQC spectrum of 1-acetyl-sphaerocarpain I (2)** **in CDCl_3_.**


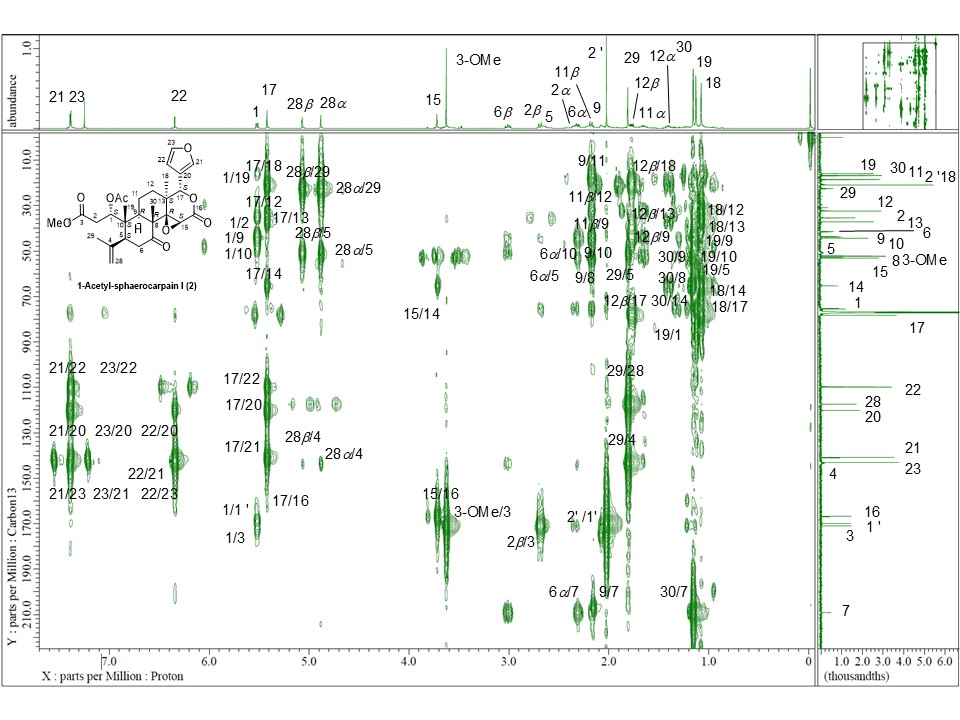

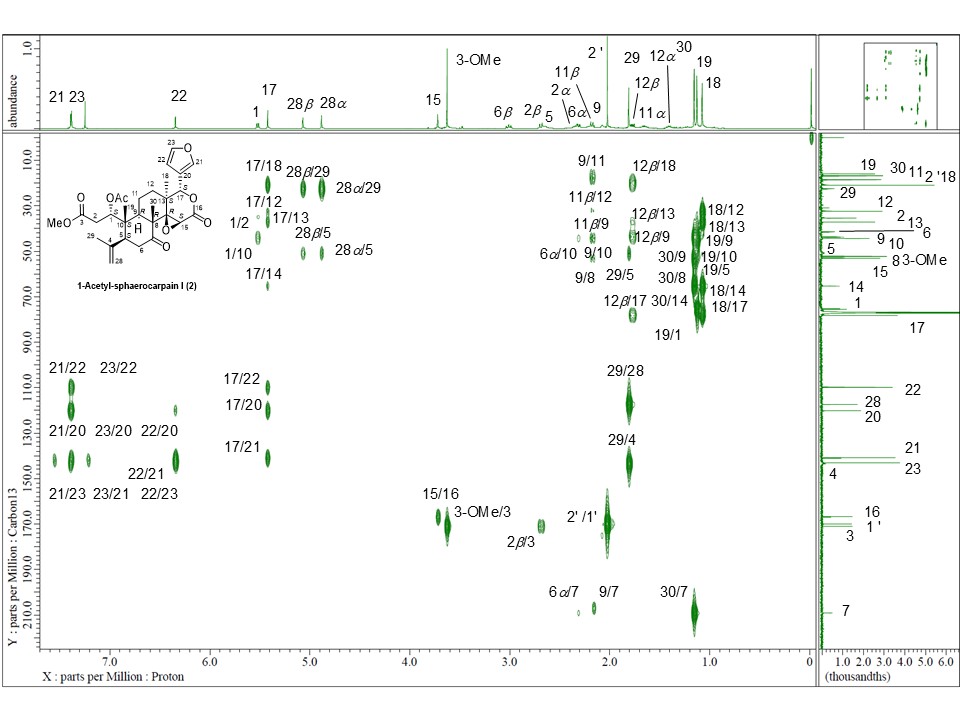


**Figure S2.4.1. HMBC spectrum of 1-acetyl-sphaerocarpain I (2)** **in CDCl_3_.**

**Figure S2.4.2. HMBC spectrum of 1-acetyl-sphaerocarpain I (2)** **in CDCl_3_.**


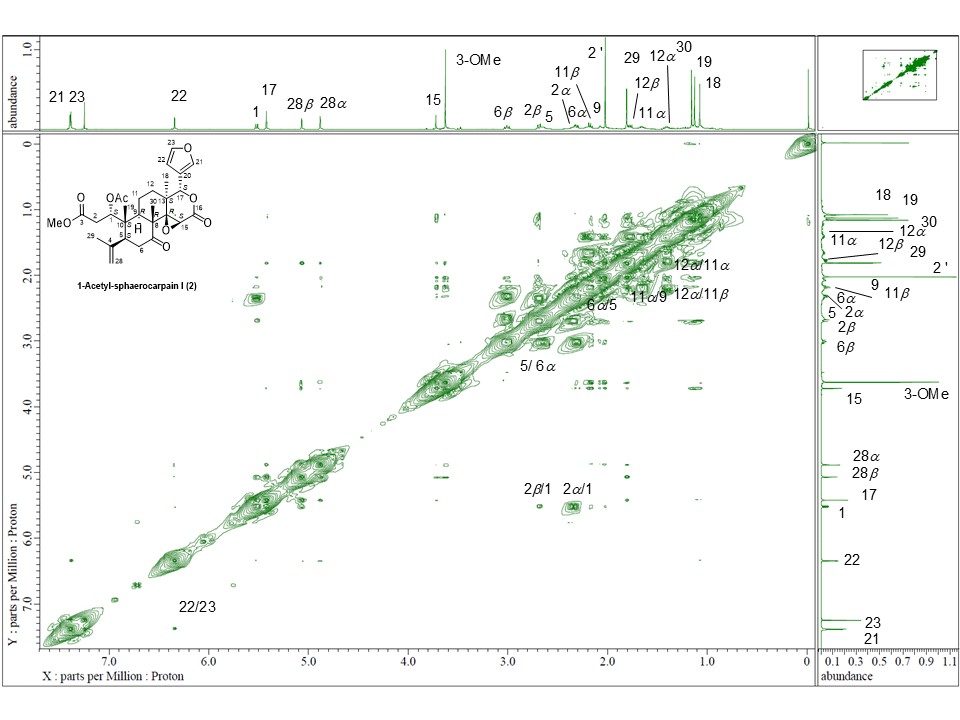


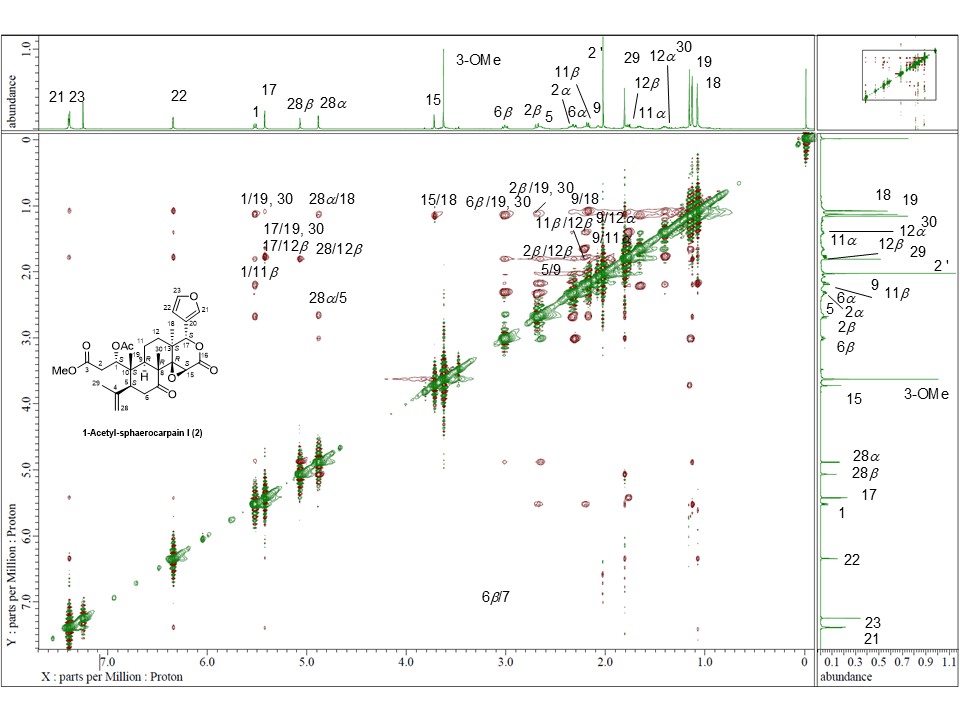
**Figure S2.5. COSY spectrum of 1-acetyl-sphaerocarpain I (2)** **in CDCl_3_.**

**Figure S2.6. NOESY spectrum of 1-acetyl-sphaerocarpain I (2)** **in CDCl_3_.**


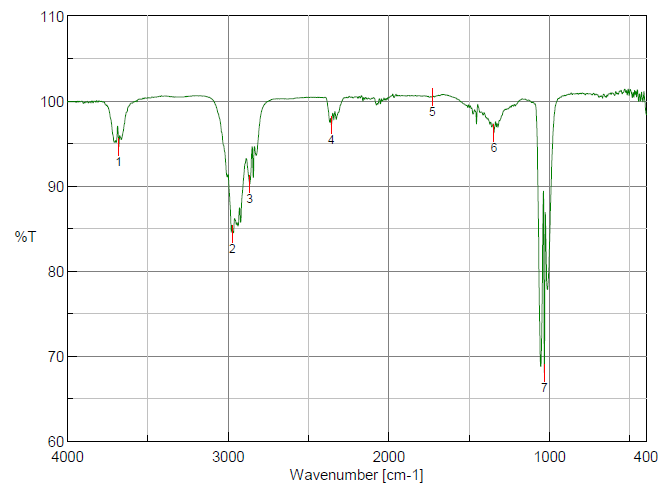


**Figure S2.7. IR spectra of 1-acetyl-sphaerocarpain I (2).**


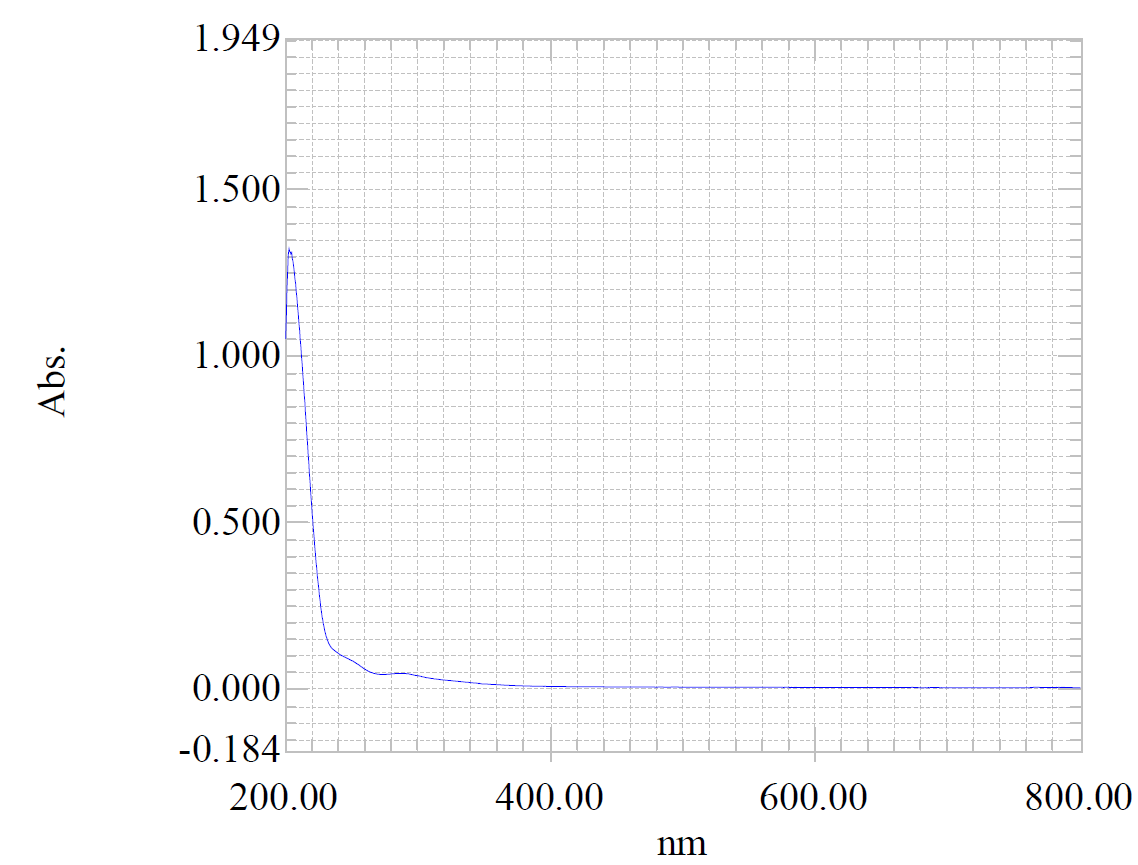


**Figure S2.8. UV spectra of 1-acetyl-sphaerocarpain I (2).**


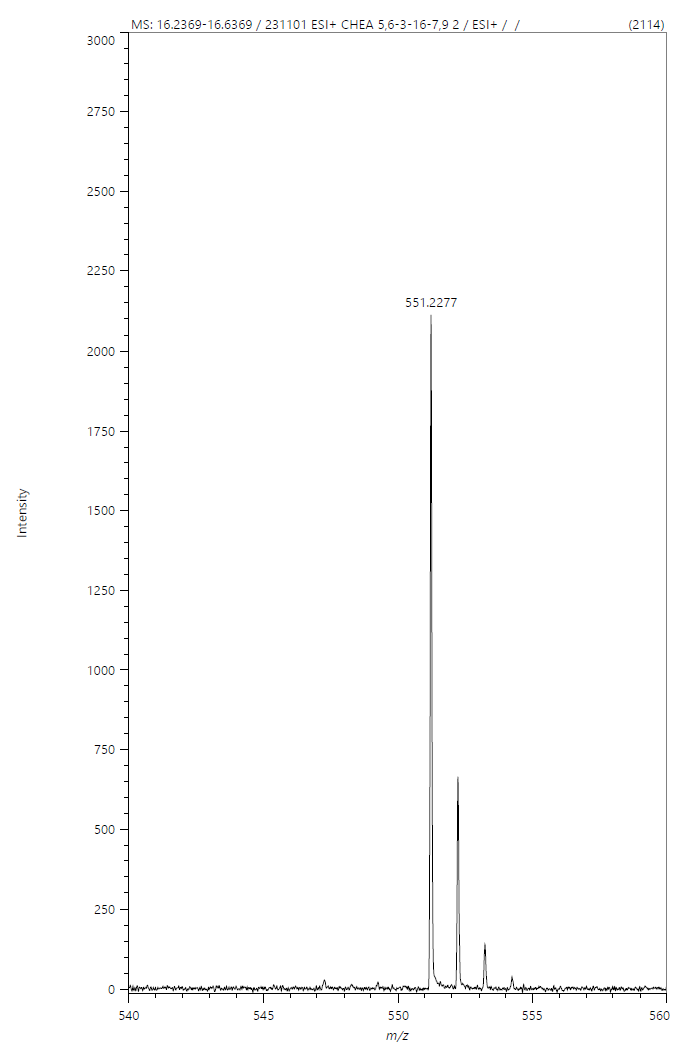


**Figure S2.9. MS spectra of 1-acetyl-sphaerocarpain I (2).**

**
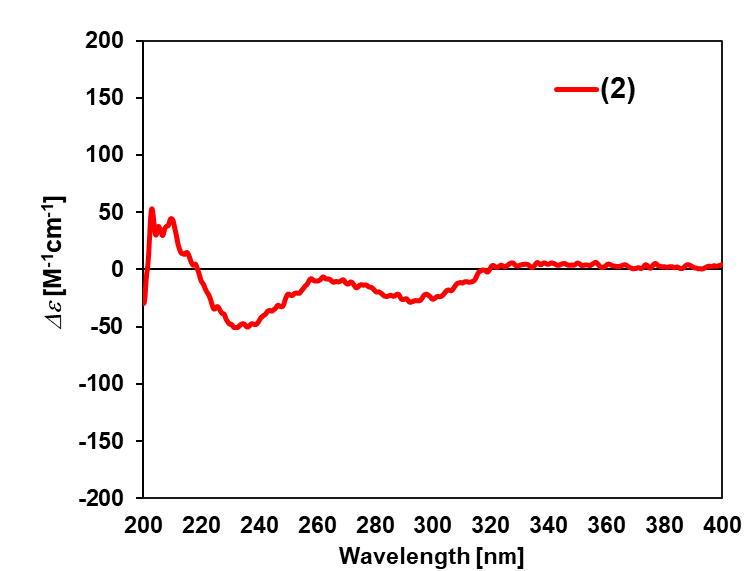
**

**Figure S2.10. CD spectra of 1-acetyl-sphaerocarpain I (2).**

**Figure S2.11. Skeletal (shorthand) structures of 1-acetyl-sphaerocarpainic acid I (1) and 1-acetyl-sphaerocarpain I (2), limonoate A-ring lactone^33^, and nomilinoate A-ring lactone^33^.**

**Figure S2.12. Synthetic Procedure for the Conversion of Compound 2 to Methyl Nomilinate (5) via Mukaiyama Hydration.**

To a solution of 1-acetyl-sphaerocarpain I (**2**) (2.0 mg, 3.79 mmol) in MeOH (0.3 ml) was added Mn(dpm)_3_ (0.16 mg, 0.26 mmol) and PhSiH_3_ (0.8 μL, 7.39 mmol) under O_2_ atmosphere, and the mixture was stirred for 0.5 h. The reaction mixture was quenched with sat. Na_2_S_2_O_3_ aqueous solution and extracted with AcOEt three times. The combined organic layers were washed with brine, dried over MgSO_4_, and evaporated under reduced pressure. The resulting residue was purified by HPLC [H_2_O/MeCN/AcOH (70:30:0.3)] to afford methyl nomilinate (**5**) (1.8 mg, 3.3 mmol, 90%). ${[a]}_{D}^{25}$= –47.8 (c 0.04, MeOH); ^1^H NMR (600 MHz, DMSO-d_6_) δ_H_ 6.42 (1H, br s, H-1), 2.88 (1H, t, J= 12.6 Hz, H-2), 2.32 (2H, m, H-2/H-11), 2.03 (1H, d, J=12.0 Hz, H-5), 3.00 (1H, d, J= 15.0 Hz, H-6), 2.22 (1H, t, J=11.4 Hz, H-6), 2.07 (1H, d, J=12.0 Hz, H-9), 1.52–1.55 (1H, m, H-11), 1.78–1.81 (1H, m, H-12), 1.97 (4H, br s, OAc-1/H-12), 3.76　(1H, s, H-15), 5.43 (1H, s, H-17), 1.01 (3H, s, H-18), 1.06 (3H, s, H-19), 7.67 (1H, s, H-21), 6.53 (1H, s, H-22), 7.74 (1H, s, H-23) , 1.22 (6H, s, H-28/H-29), 1.19 (3H, s, H-30), 3.51 (3H, s, OMe-3); ^13^C NMR (150 MHz, DMSO-d_6_) δ_C_ 209.8, 171.2, 169.7, 167.2, 143.4, 141.5, 120.1, 110.2, 77.6, 75.8, 72.9, 65.3, 52.3, 52.0, 52.0, 51.4, 45.5, 43.7, 38.4, 36.5, 34.5, 32.7, 31.2, 28.2, 20.9, 20.4, 18.3, 16.1, 15.9; HR-ESI-MS Calcd for C_29_H_38_O_10_Na [M+Na]^+^ 569.2357. Found 569.2353.


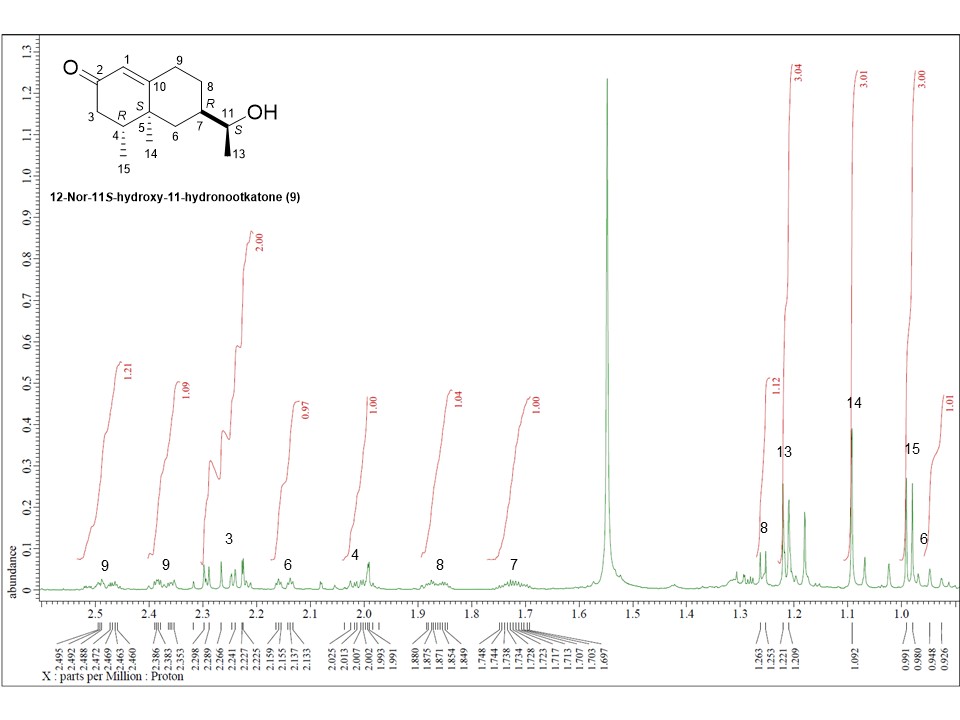

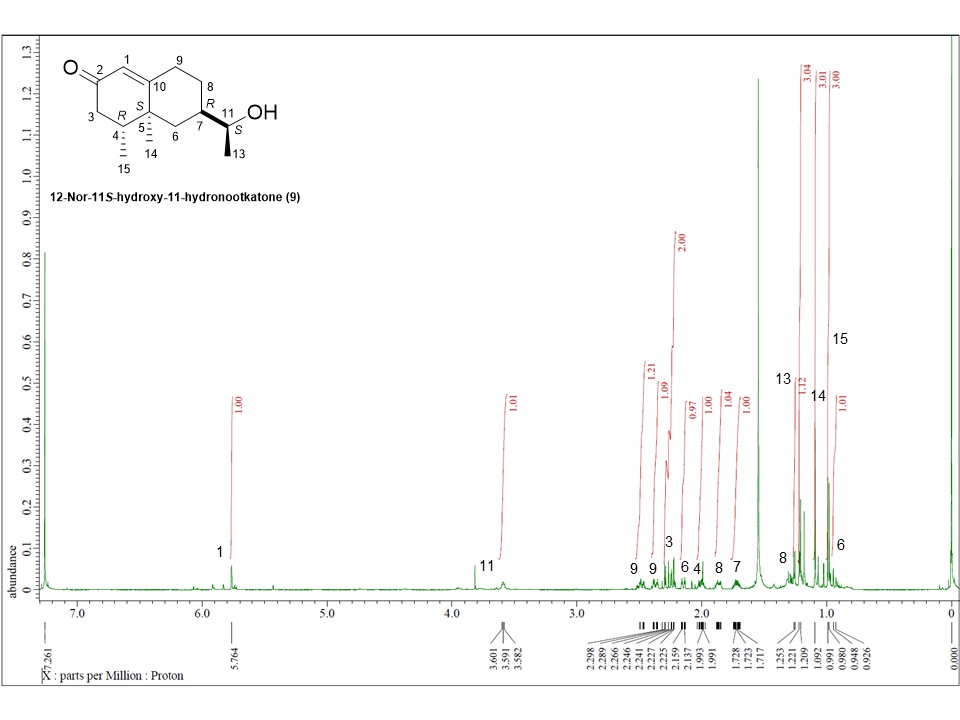
**Figure S3.1.1. ^1^H NMR spectrum of 12-nor-11S-hydroxy-11-hydronootkatone (9)** **in CDCl_3_.**

**Figure S3.1.2. ^1^H NMR spectrum of 12-nor-11S-hydroxy-11-hydronootkatone (9)** **in CDCl_3_.**


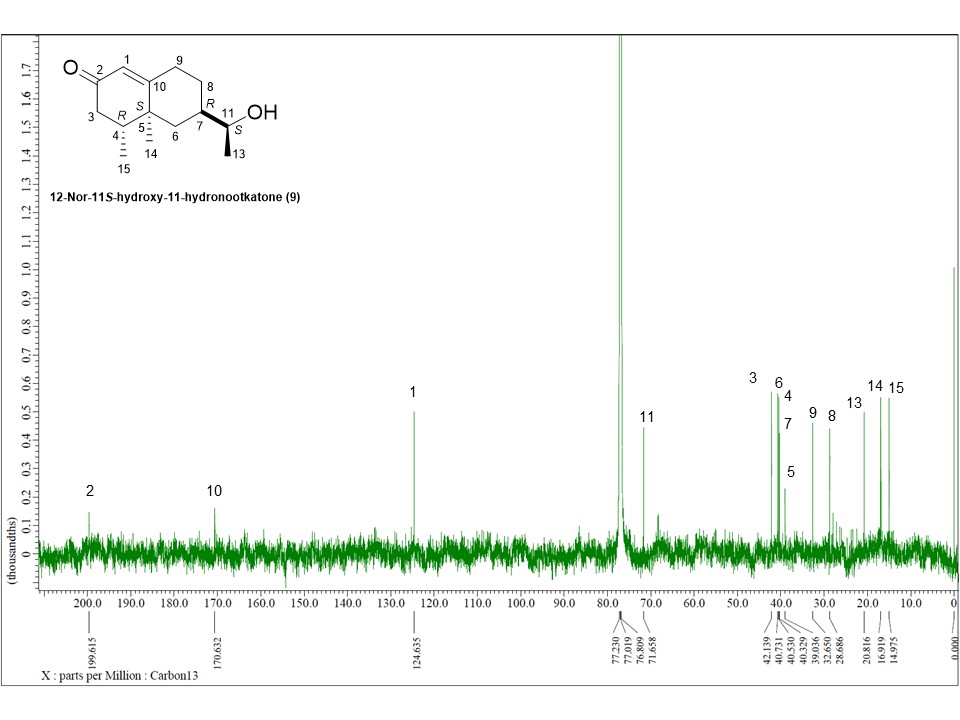
**Figure S3.2. ^13^C NMR spectrum of 12-nor-11S-hydroxy-11-hydronootkatone (9)** **in CDCl_3_.**


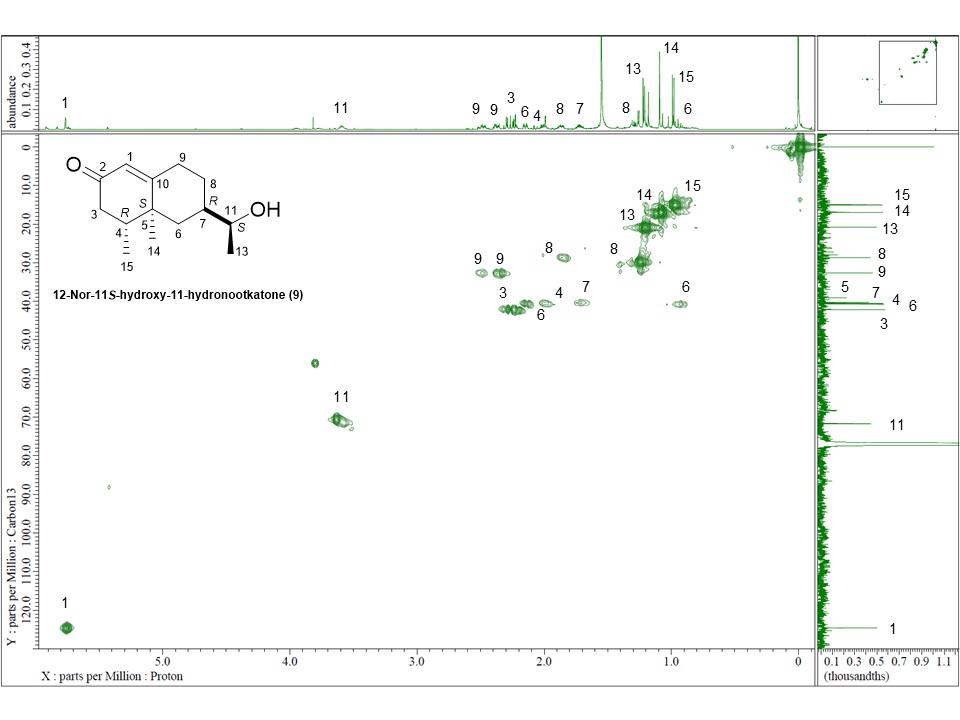


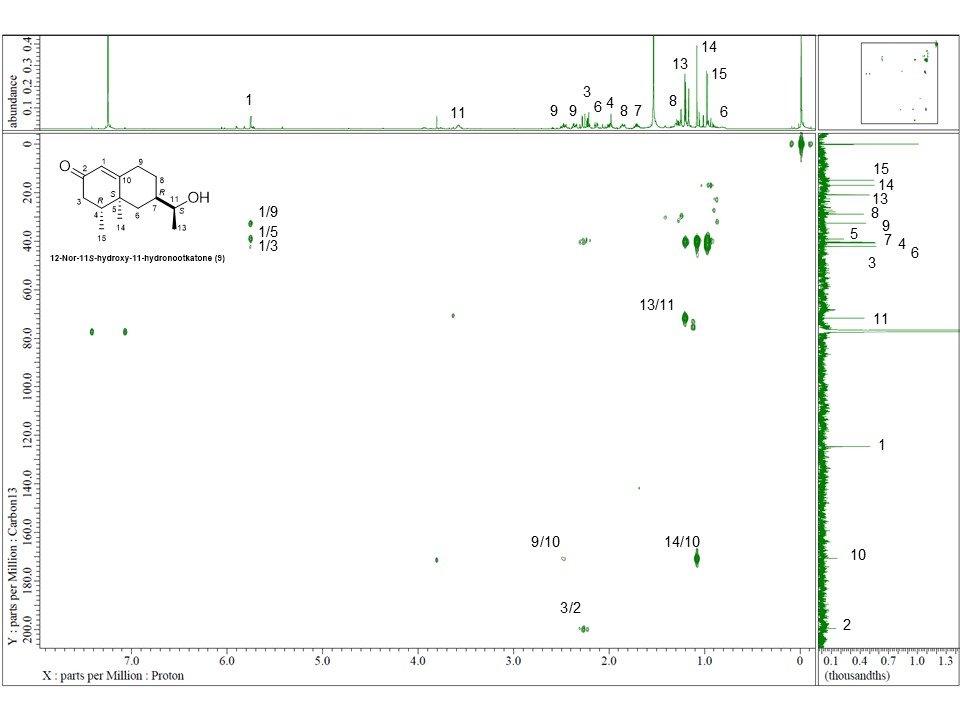
**Figure S3.3. HMQC spectrum of 12-nor-11S-hydroxy-11-hydronootkatone (9).**

**Figure S3.4.1. HMBC spectrum of 12-nor-11S-hydroxy-11-hydronootkatone (9)** **in CDCl_3_.**


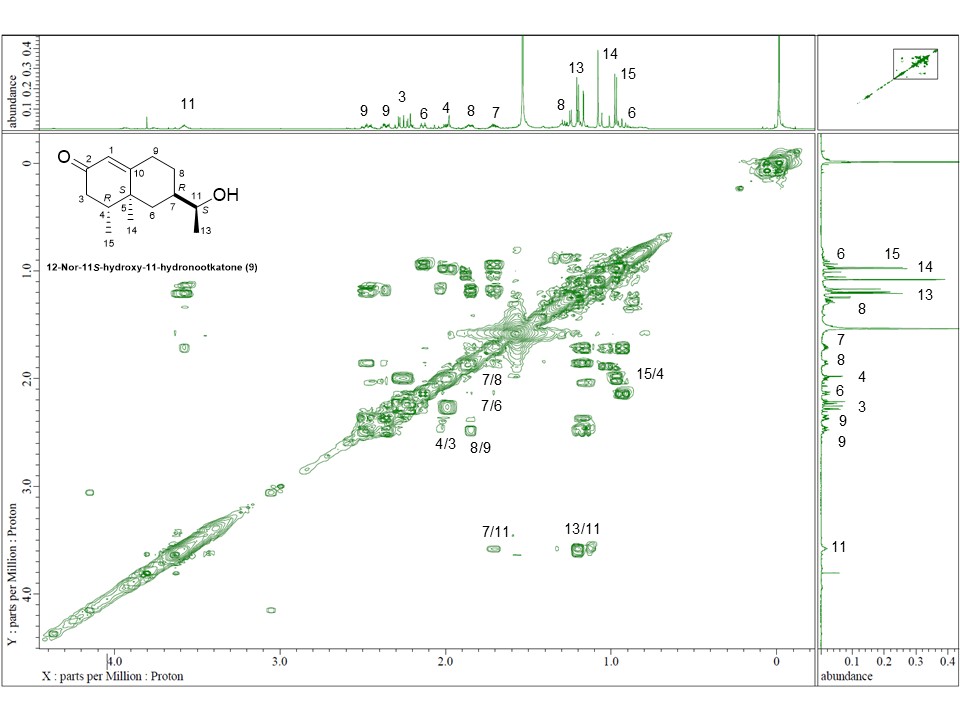

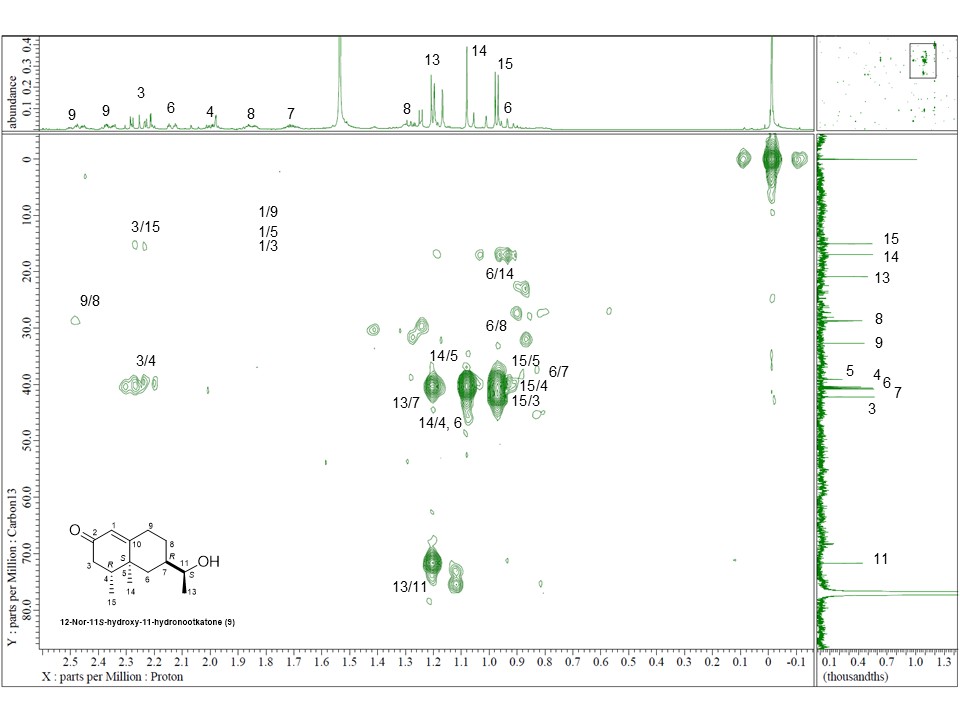
**Figure S3.4.2. HMBC spectrum of 12-nor-11S-hydroxy-11-hydronootkatone (9)** **in CDCl_3_.**

**Figure S3.5. COSY spectrum of 12-nor-11S-hydroxy-11-hydronootkatone (9)** **in CDCl_3_.**

**
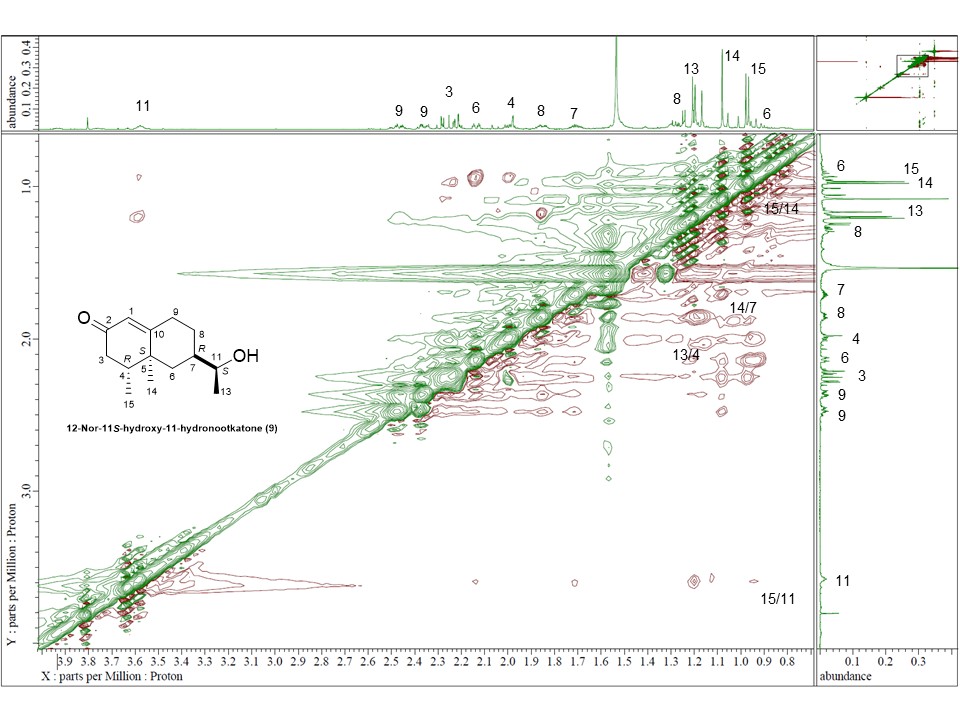
Figure S3.6. NOESY spectrum of 12-nor-11S-hydroxy-11-hydronootkatone (9)** **in CDCl_3_.**


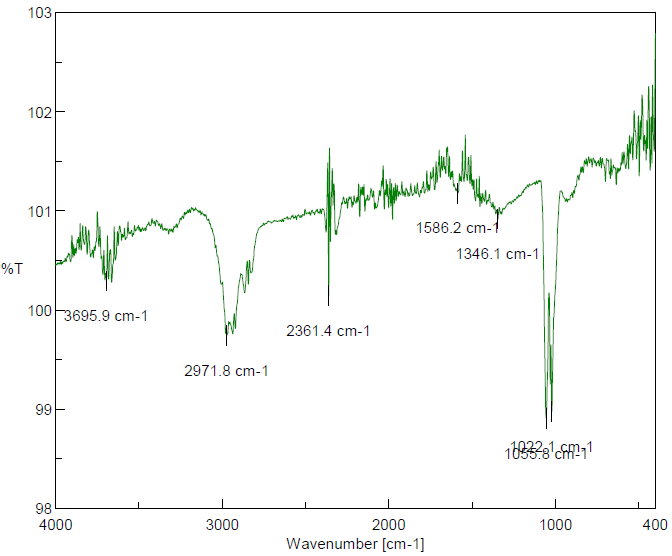


**Figure S3.7. IR spectra of 12-nor-11S-hydroxy-11-hydronootkatone (9).**


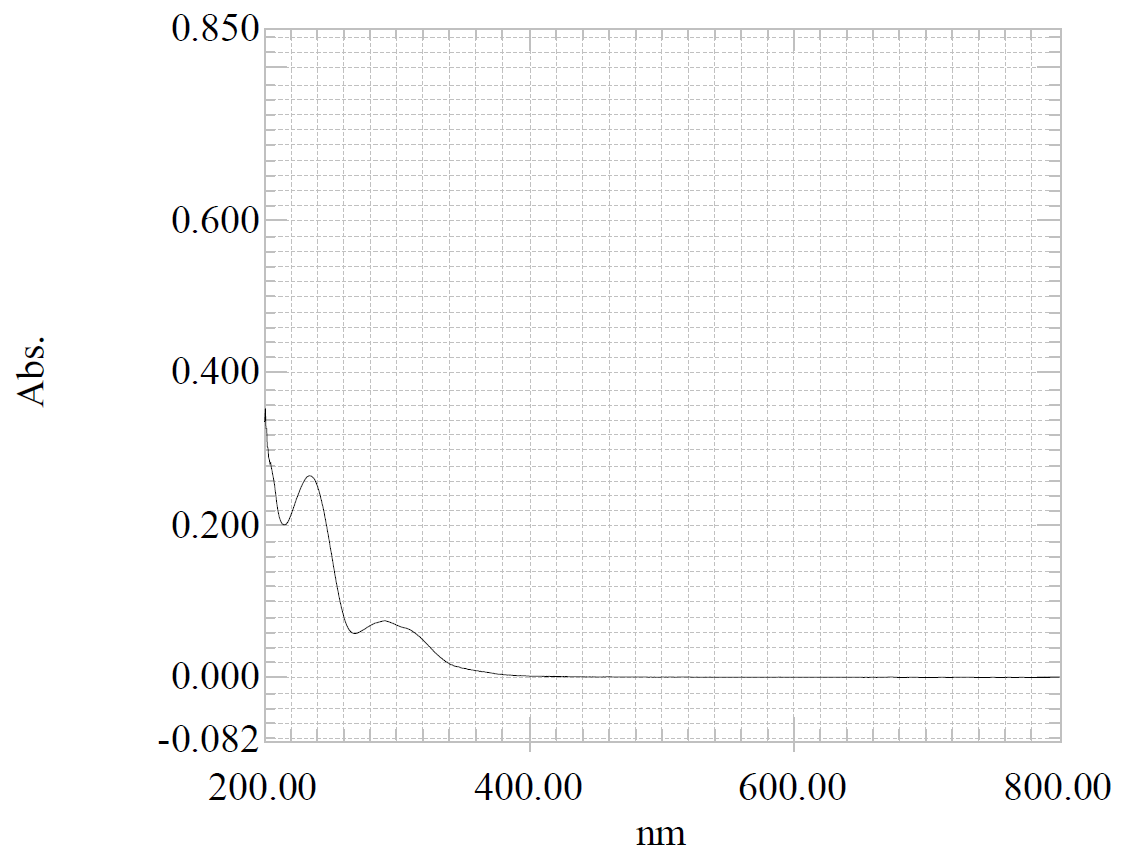


**Figure S3.8. UV spectra of 12-nor-11S-hydroxy-11-hydronootkatone (9).**


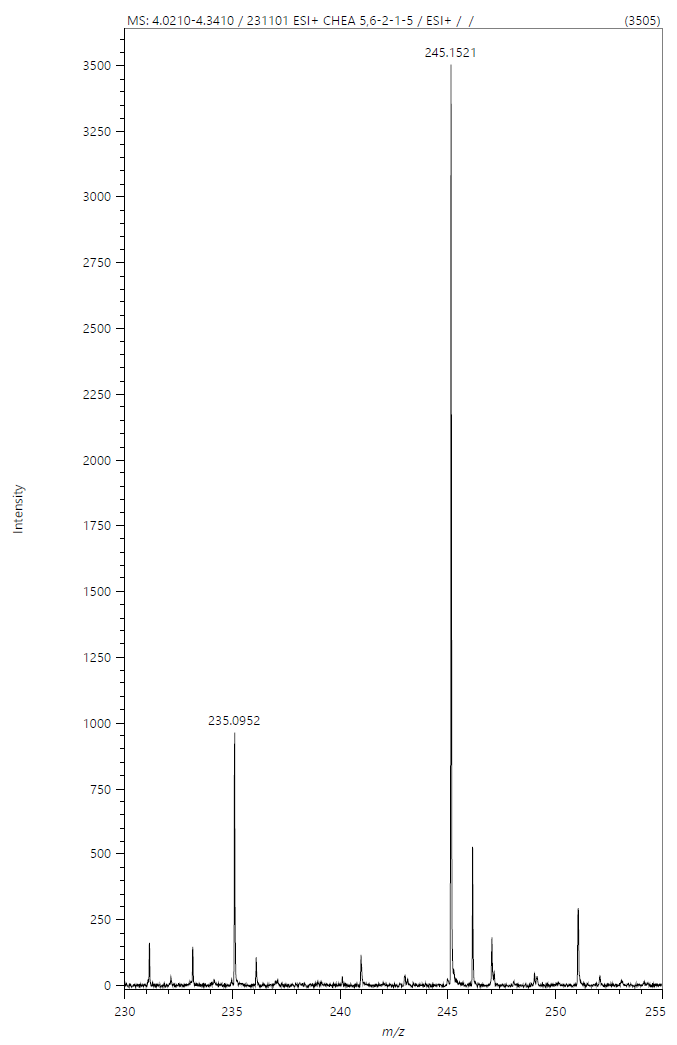


**Figure S3.9. MS spectra of 12-nor-11S-hydroxy-11-hydronootkatone (9).**


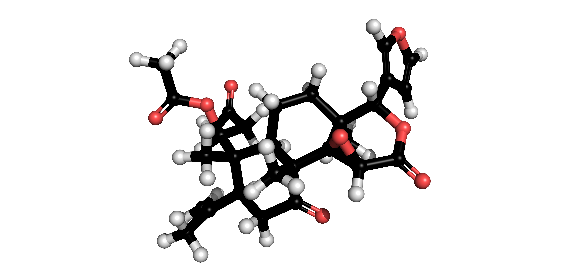

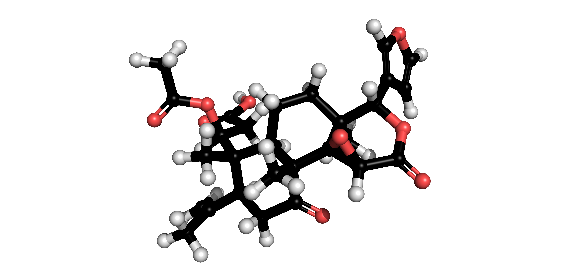

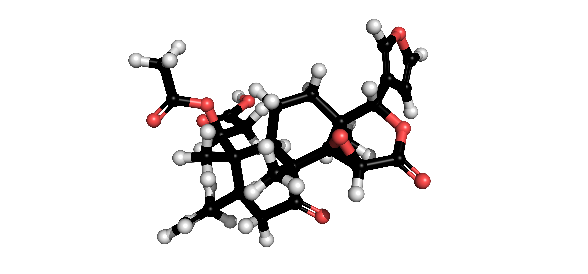

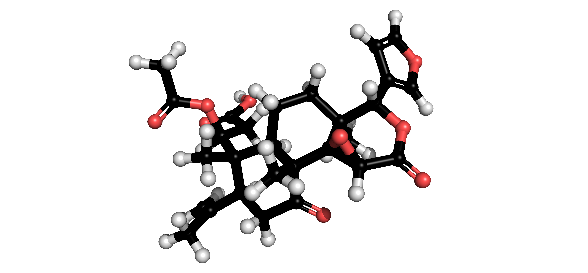


**Conf. 3**[24.79]
*ΔH* = 0.44, P = 10.13

**Conf.1**[22.47]
*ΔH* = 0.00, P = 21.24

**Conf. 2**[26.43]
*ΔH* = 0.16, P = 16.3

**Conf. 4**[23.83]
*ΔH* = 0.46, P = 9.70


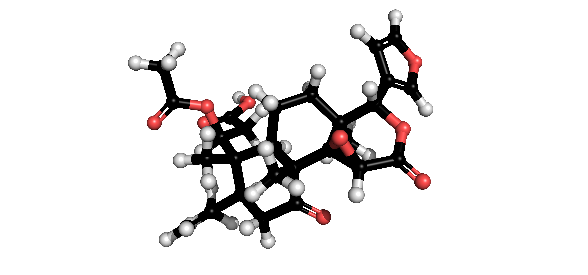

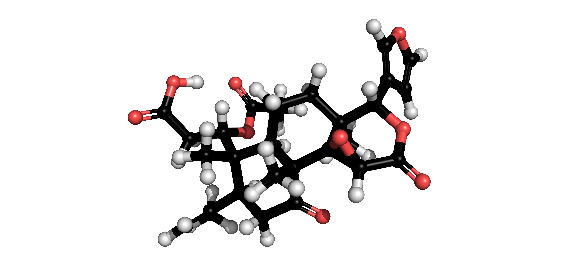

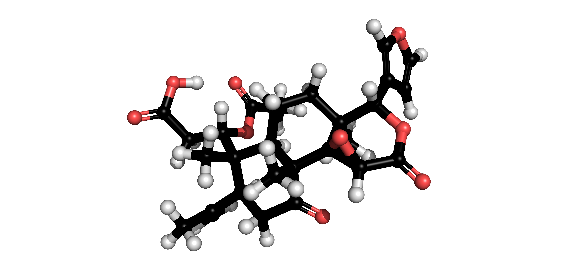

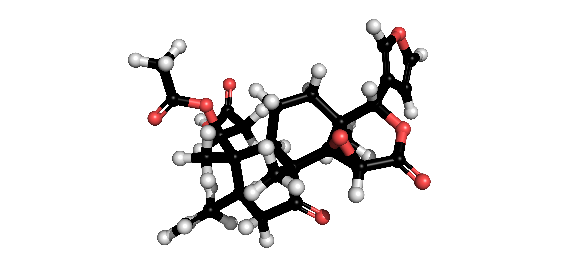


**Conf. 6**[22.89]
*ΔH* = 0.67, P = 6.82

**Conf. 7**[6.81]
*ΔH* = 0.74, P = 6.07

**Conf. 5**[4.48]
*ΔH* = 0.60, P = 7.77

**Conf. 8**[24.45]
*ΔH* = 0.80, P = 5.46


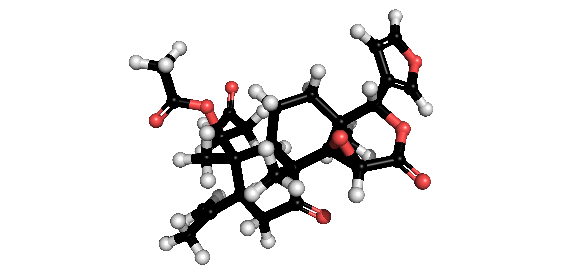

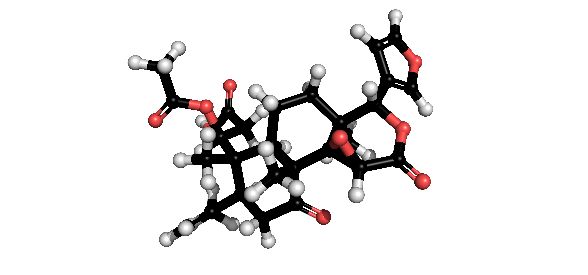

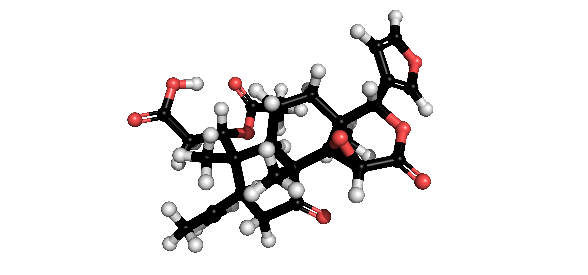

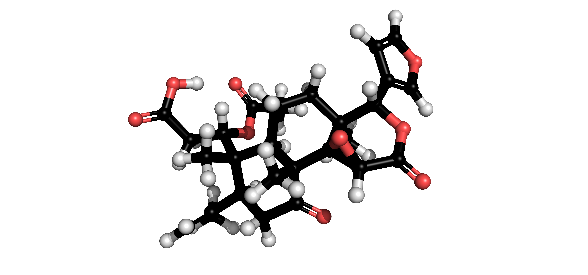


**Conf. 9**[23.75]
*ΔH* = 0.88, P = 4.83

**Conf. 11**[21.15]
*ΔH* = 1.32, P = 2.29

**Conf. 10**[25.21]
*ΔH* = 1.27, P = 2.49

**Conf. 12**[24.47]
*ΔH* = 1.33, P = 2.26


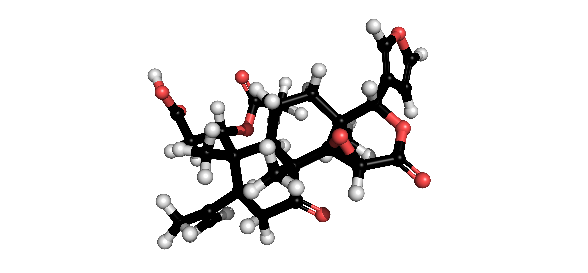

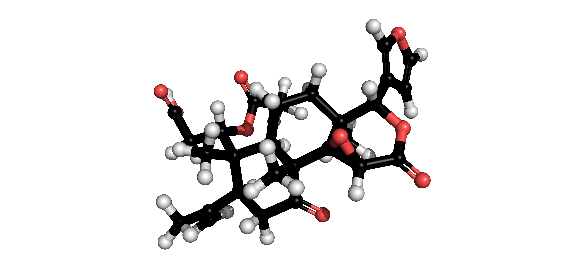


**Conf. 13**[24.67]
*ΔH* = 1.67, P = 1.27

**Conf. 14**[20.48]
*ΔH* = 1.78, P = 1.06

Figure S4. Optimized geometries, the minimum value of frequency, relative enthalpies including the ZPE correction, and Boltzmann distributions of conformers of 1-acetyl-sphaerocarpainic acid I (1).

Figure S4. The optimized structures of 14 conformers of **1** with the minimum value of frequency [in brackets, cm^−1^], relative enthalpies including the ZPE correction (*ΔH*, kcal/mol), and Boltzmann distribution (P, %), at 298.15 K, calculated at the *ω*B97X-D/def2-TZVP level in MeOH.


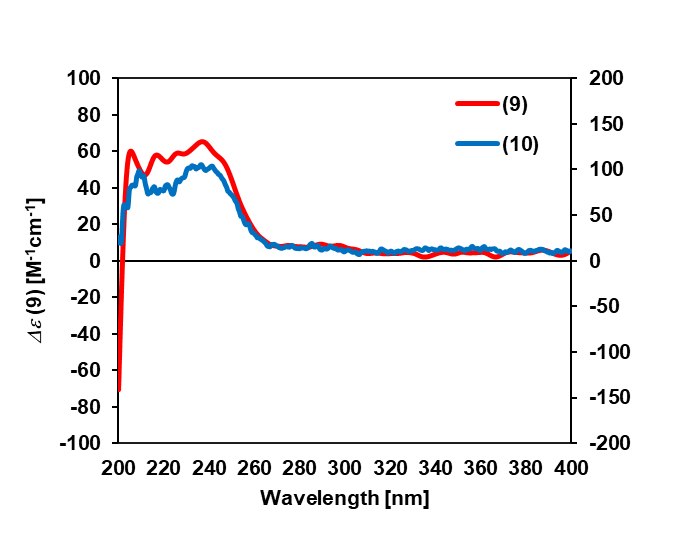

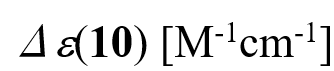


Figure S5.1. CD spectra of 12-nor-11*S*-hydroxy-11-hydronootkatone (9) and nootkatone (10).


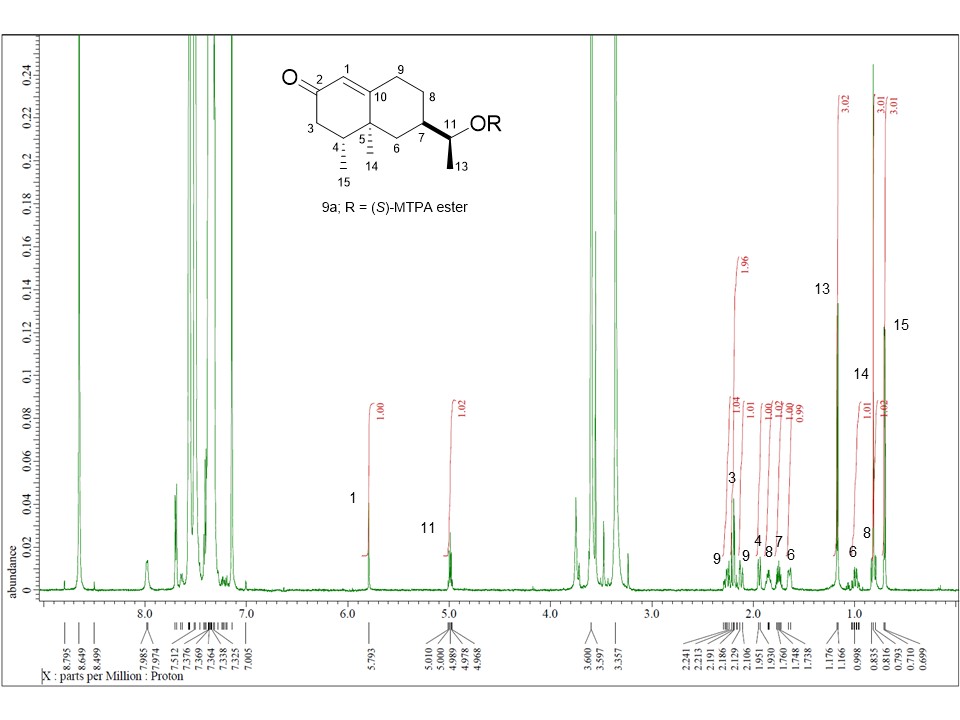


Figure S5.2. ^1^H NMR spectrum of 9a in pyridine-*d*_5_.


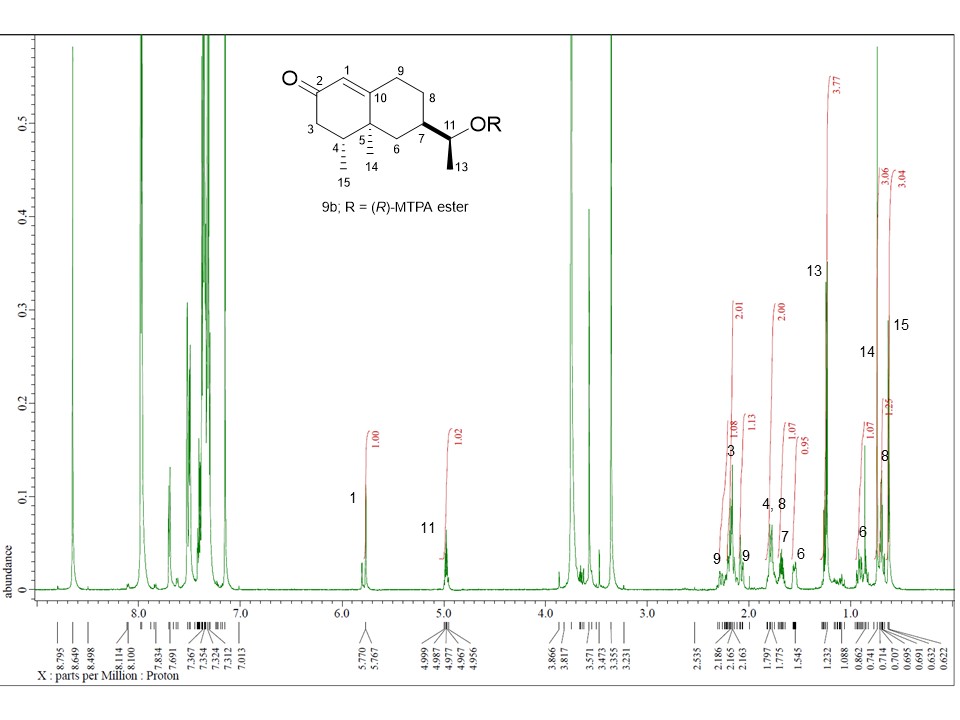


Figure S5.3. ^1^H NMR spectrum of 9b in pyridine-*d*_5_.


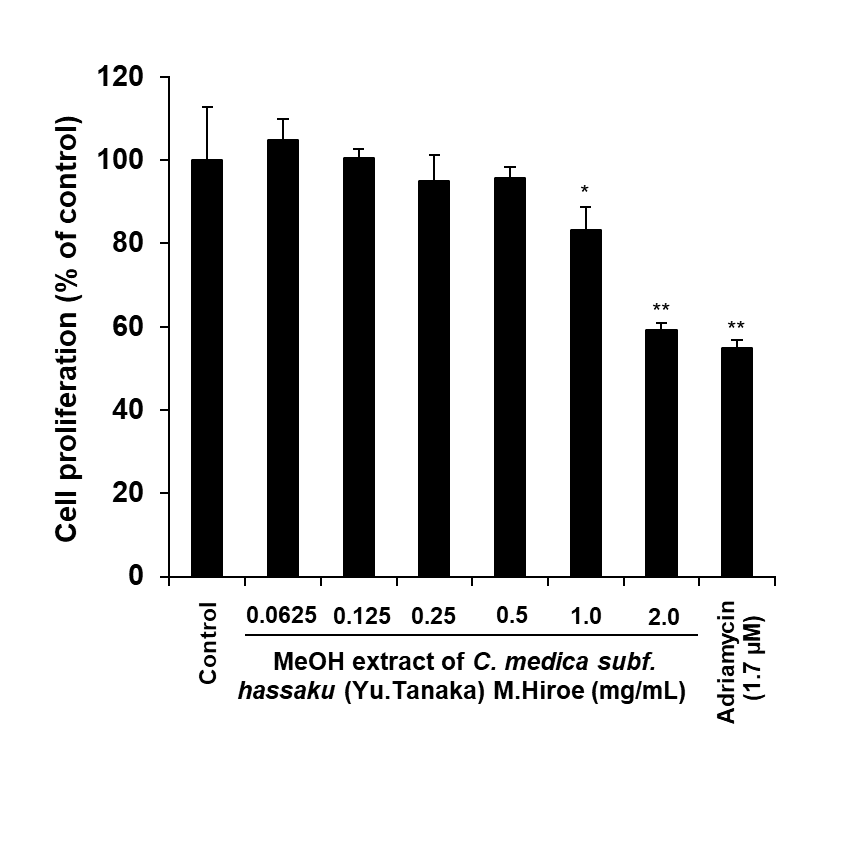


Figure S6. Anti-proliferative effects evaluation of adriamycin by WST-8 assay.

Figure S6. U-251 MG cells were seeded into 96-well plates (2,500 cells/well) and cultured at 37 °C. After 24 h, MeOH extract of *C. hassaku* Yu.Tanaka or adriamycin (1.7 µM) added to the culture medium at MeOH extract concentrations of 0.0625, 0.125, 0.25, 0.5, 1.0, 2.0 mg/mL, and cells were cultured at 37 °C for 24 h. Surviving cells were detected by WST-8 assay. Data reported as the mean ± SD of three independent experiments. Statistical significance was analyzed using the Dunnett's multiple comparisons test (^⁎^*P*<0.05, or ^⁎⁎^*P*<0.01 compared with DMSO-treated cells). The cell proliferation rates of U-251 MG cells for adriamycin (1.7 µM) were 55.0 ± 1.88%.


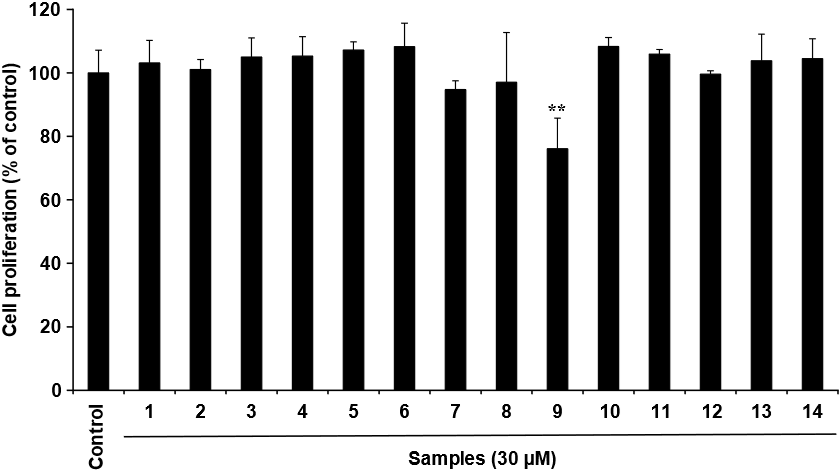


Figure S7. Anti-proliferative effects evaluation of the isolated compounds (1–14) by WST-8 assay.

Figure S7. U-251 MG cells were seeded into 96-well plates (2,500 cells/well) and cultured at 37 °C. After 24 h, isolated compounds (**1**–**14**) were added to the culture medium at concentrations of 30 μM, and cells were cultured at 37 °C for 24 h. Surviving cells were detected by WST-8 assay. Data reported as the mean ± SD of three independent experiments. Statistical significance was analyzed using Dunnett’s multiple comparison test (^⁎⁎^*P*<0.01 compared with DMSO-treated cells). Adriamycin was used as a positive control.

**Table S1. ^13^C (100 MHz) and ^1^H (400 MHz) NMR (in methanol-*d*_3_) data for limonoate A-ring lactone^33^ and nomilinoate A-ring lactone^33^.**

|  | **limonoate A-ring lactone**^33^ | | **nomilinoate A-ring lactone**^33^ | |
| --- | --- | --- | --- | --- |
| Position | *δ* _C_ | *δ* _H_ (*J* in Hz) | *δ* _C_ | *δ* _H_ (*J* in Hz) |
| 1 | 79.6 | 4.30 (1H, s) | 72.5 | 4.76 (1H, d, 8.0) |
| 2 | 36.9 | 2.82 (2H, s) | 36.4 | *a* 3.37 (1H, d, 16.0)  *b* 3.13 (1H, dd, 16.0, 8.0) |
| 3 | 175.7 |  | 175.4 |  |
| 4 | 82.1 |  | 86.7 |  |
| 5 | 62.3 | 2.57 (1H, dd, 19.2, 5.6) | 48.0 | 3.11 (1H, dd, 16.0, 8.4) |
| 6 | 38.0 | *a* 2.93 (1H, dd, 5.6, 14.8)  *b* 2.67 (1H, d, 14.8) | 41.8 | *a* 2.14 (1H, dd, 8.4, 18.8)  *b* 3.04 (1H, dd, 8.4, 18.8) |
| 7 | 211.1 |  | 213.7 |  |
| 8 | 53.2 |  | 53.5 |  |
| 9 | 47.2 | 2.71–2.77 (1H, m) | 44.2 | 2.74 (1H, dd, 12.4, 7.6) |
| 10 | 47.2 |  | 45.1 |  |
| 11 | 19.0 | *a* 1.75–1.85 (1H, m)  *b* 1.87–1.99 (1H, m) | 17.2 | *a* 1.58 (1H, m, 12.4)  *b* 1.35 (1H, m) |
| 12 | 31.8 | *a* 1.87–1.99 (1H, m)  *b* 1.25–1.40 (1H, m) | 31.7 | *a* 1.83–1.92 (1H, m)  *b* 1.17–1.24 (1H, m) |
| 13 | 45.6 |  | 44.5 |  |
| 14 | 72.9 |  | 73.0 |  |
| 15 | 56.1 | 2.96 (1H, s) | 62.5 | 2.85 (1H, s) |
| 16 | 173.7 |  | 171.5 |  |
| 17 | 72.5 | 5.13 (1H, s) | 72.5 | 5.09 (1H, s) |
| 18 | 20.3 | 1.09 (3H, s) | 20.7 | 1.23 (3H, s) |
| 19 | 65.6 | *a* 4.48 (1H, d, 13.2)  *b* 4.54 (1H, d, 13.2) | 14.1 | 1.35 (3H, s) |
| 20 | 127.9 |  | 127.9 |  |
| 21 | 142.8 | 7.55 (1H, s) | 142.8 | 7.54 (1H, s) |
| 22 | 142.9 | 6.50 (1H, s) | 142.9 | 6.49 (1H, s) |
| 23 | 112.2 | 7.37 (1H, s) | 112.1 | 7.36 (1H, s) |
| 28 | 30.6 | 1.32 (3H, s) | 32.7 | 1.41 (3H, s) |
| 29 | 23.2 | 1.33 (3H, s) | 23.1 | 1.52 (3H, s) |
| 30 | 22.3 | 1.07 (3H, s) | 20.9 | 1.02 (3H, s) |
| -OAc |  |  | 22.2 | 2.01 (3H, s) |
|  |  |  | 173.2 |  |
